# TWISTER (Twente water injection system for turbulence experimental research): a jet array in the Twente water tunnel for generating strong turbulence using four-dimensional gradient noise

**DOI:** 10.1007/s00348-025-04103-z

**Published:** 2025-09-14

**Authors:** Duco van Buuren, Dennis P. M. van Gils, Gert-Wim Bruggert, Dominik Krug

**Affiliations:** 1 Physics of Fluids Group, J. M. Burgers Center for Fluid Dynamics and Max Planck Center Twente, Faculty of Science and Technology, University of Twente, P.O. Box 217, 7500 AE Enschede, The Netherlands; 2 Institute of Aerodynamics and Chair of Fluid Mechanics, Faculty of Mechanical Engineering, RWTH Aachen University, Wüllnerstrasse 5a, 52062 Aachen, Germany

## Abstract

We present a newly constructed jet array with a novel driving scheme for turbulence generation in a vertical water tunnel and measurements of the turbulent flow this jet array establishes. The design of the array allows us to control the mean background flow and the turbulence intensity independently of each other. The array consists of a rectangular arrangement of 112 individually computer-controlled water jets that are aligned streamwise to the measurement section of our 8-m tall vertically recirculating water tunnel. Using solenoid valves, individual jets are activated following predefined protocols that can be tailored to obtain different turbulence statistics within the measurement section. The protocols are based on four-dimensional OpenSimplex noise, a type of gradient noise that features spatial and temporal coherence. Details of the mechanical and electrical designs are presented, together with a detailed description of the protocol generation. We show that the resulting turbulence is near homogeneous and isotropic, with a turbulence intensity of Order 1, an energy dissipation rate of order $10^{-1}\,\mathrm {m^2/s^3}$ and $\textrm{Re}_{\lambda }\approx 1400$. Additionally, we present experiments that show the effects that various system and protocol parameters have on the created flow conditions and address the streamwise development, as well as the homogeneity and isotropy of the flow.

## Introduction

Multiphase turbulent flow occurs all throughout nature and industry, with examples including oceanic and coastal flows, as well as combustion processes. To study such flows, experimental setups have been built utilizing numerous methods for the creation of turbulence, such as propellers (Zimmermann et al. 2010), active grids (Poorte and Biesheuvel 2002; Makita 1991; Bodenschatz et al. 2014; Neuhaus et al. 2020), jet-based systems (Gad-el Hak and Corrsin 1974; Variano et al. 2004b; Carter et al. 2016; Masuk et al. 2019; McCutchan and Johnson 2023; Ghazi Nezami and Johnson 2024), and rotating disks (Lachize et al. 2016). Our intended future multiphase research focus is on bubble-particle collisions in turbulence. This is a topic directly influenced by an example of multiphase flow in industry, namely a froth flotation cell. In this large system, a bath of water is stirred, and bubbles and particles are added in an effort to separate different species of material. In this example many questions remain regarding the effect that turbulence has on the small-scale interactions between the bubbles and particles. Vertical water tunnel setups are well suited for studying the behavior of bubbles in turbulence since they can have a flow counter to the rising bubbles, such that the bubble residence time in the measurement volume is increased.

Historically, water tunnels have employed passive (Comte-Bellot and Corrsin 1966) or active grids (Poorte and Biesheuvel 2002; Makita 1991) as turbulence creation methods. However, the turbulence strength and intensity achievable with these systems often falls short of what can be found in practical applications. Additionally, these systems rely mainly or fully on the mean (background) flow to supply the energy from which the turbulence is created. Therefore, a large mean flow is required to achieve powerful turbulence. This leads to an issue for research on rising bubbles in strong turbulence since a large mean flow is required for the strong turbulence, but a small mean flow is desired for long residence times. Our goal is therefore to devise a method through which turbulence parameters can be controlled (largely) independently of the mean flow.

To have strong turbulence and a small mean flow, the method of turbulence creation and the background flow have to be separated. A logical choice for our system is, therefore, to use jets. This method has been applied before, notably by Gad-el Hak and Corrsin (1974); Variano et al. (2004a); Bellani and Variano (2014); Carter et al. (2016); Pérez-Alvarado et al. (2016); Pratt et al. (2017); Masuk et al. (2019); McCutchan and Johnson (2023); Bang and Pujara (2023), and Ghazi Nezami and Johnson (2024). In these systems, the jets are either all active continuously or their intermittent firing sequence is based on some specific protocol, often the so-called sunbathing algorithm (Variano et al. 2004a). In that protocol there are pre-determined, normally distributed probabilities for the "on" and "off" times of every individual jet. That means that there is no spatial coherence in the sunbathing protocol. (Pérez-Alvarado et al. 2016) have shown that this lack of spatial coherence benefits the turbulence characteristics, in cases where the distribution of active jets is approximately homogeneous. Protocols of the "sunbathing" kind can be tailored to ensure a certain average fraction of active jets over time, which (Variano et al. 2004a) denotes with $\phi$, and which we will refer to as the array transparency.

For our system it is important that the driving protocol ensures an approximately constant transparency. If fluctuations in $\phi$ become too large, which can correspond to a sudden closure of many solenoid valves, it could result in a pressure spike, causing damage to the system. To mitigate this issue, we have developed a completely new type of protocol based on OpenSimplex noise—a type of gradient noise—which we will address in detail in section 3. We have chosen to implement our new jet array in our existing flow facility, the Twente Water Tunnel. We will address the Twente Water Tunnel in section 2.1. The design of our jet array is similar to the V-ONSET facility (Masuk et al. 2019), but the differing dimensions ($0.45\,\textrm{m}\times 0.45\,\textrm{m}\times 2\,\textrm{m}$ for our test section compared to $0.28\,\textrm{m}\times 0.28\,\textrm{m}\times 0.8\,\textrm{m}$ for V-ONSET), the fact that the array was implemented in an existing system, and the requirement of fully separating the background flow and the method of turbulence creation necessitated a different construction. We will discuss the construction of the jet array in detail in section 2.2.

## Experimental flow facility

The new jet array is designed to fit into the existing Twente Water Tunnel. This facility previously housed an active grid based on a design by Makita (1991); Makita and Sassa (1991); Poorte and Biesheuvel (2002) with additional modifications made in-house over the years. While the existing grid served its purpose well, it falls short for our future research needs. Specifically we require a high turbulence intensity (defined as the ratio between the magnitude of the turbulent fluctuations and the magnitude of the mean velocity, $u'/\langle u_x\rangle$) at low and controllable mean background flow velocities. The previous active grid was unable to achieve this, as demonstrated by the highest value of the turbulence intensity reported in Alméras et al. (2017), which was 0.125 at a mean flow velocity of $0.47\,\mathrm {m/s}$. Therefore, the active grid has been replaced with the jet array as described in this manuscript.

First, we will introduce the Twente Water Tunnel facility in Sect. 2.1. Then we will go into detail concerning the mechanical and electrical construction of the new jet array in Sect. 2.2. A discussion on the driving protocol that orchestrates all jets over time and space is provided in Sect. 3.

### Twente water tunnel

**Fig. 1:**
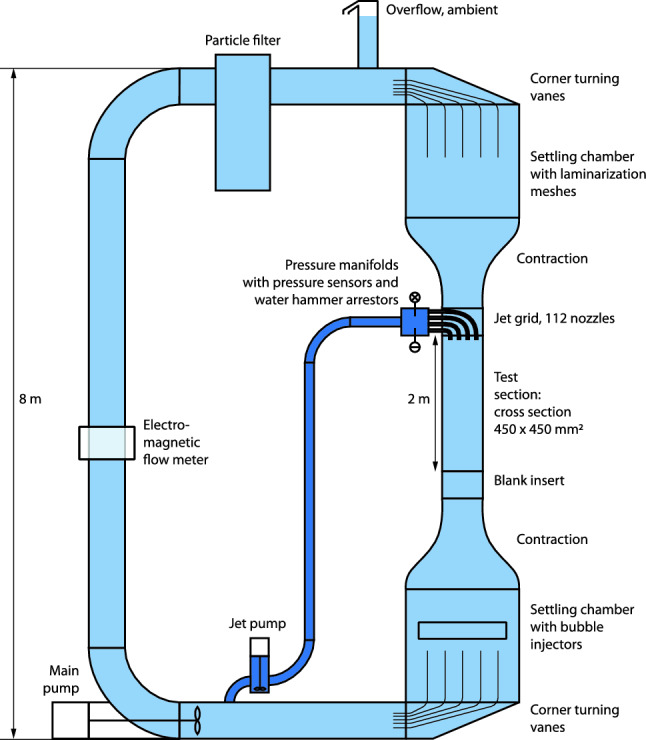
A schematic representation of the Twente Water Tunnel facility. The light-blue colored sections indicate the pre-existing facility and the dark-blue colored sections indicate the latest addition of the jet array

**Fig. 2:**
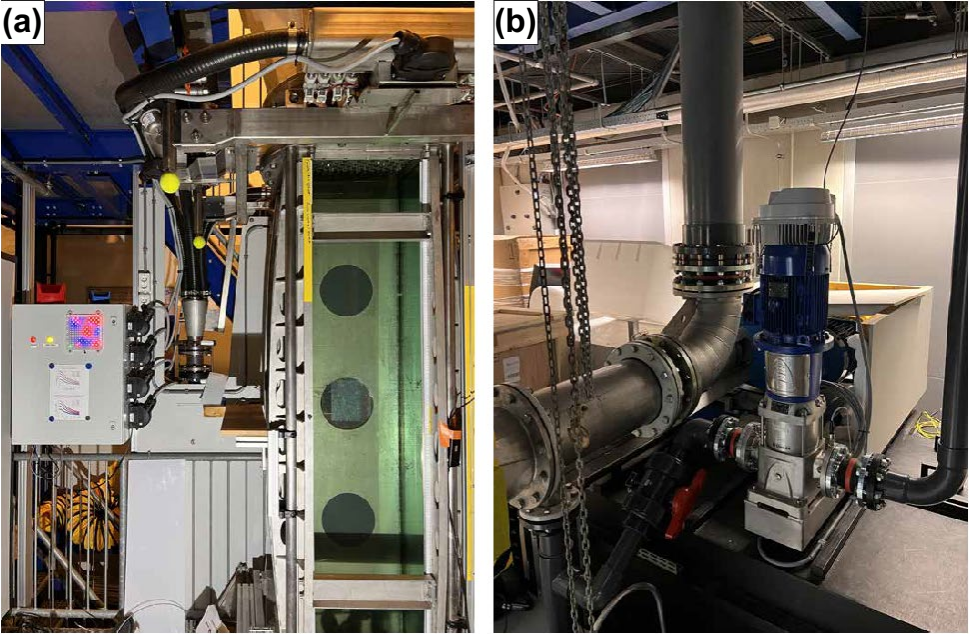
**a**: A photograph of the test section, with the round openings, named "portholes," in the stainless steel wall clearly visible. In the top of the image, part of a pressure manifold and a few connected solenoids can be seen. In the middle, the manifold that feeds the four pressure manifolds is visible, with to its left the electronics cabinet that controls the jet array. **b**: A photograph of the jet pump, with to its left the main flow loop, and to its right the pipe that goes toward the jet array

The Twente Water Tunnel facility is an 8-m tall vertical recirculating water tunnel containing 6000 ls of decalcified water, first described by Poorte and Biesheuvel (Poorte and Biesheuvel 2002) in 2002 and improved upon in later years by various other researchers. For completeness and because many of these alterations have not been published, we will provide a concise summary of the current facility here. We refer to Fig. 1 for a schematic of the tunnel. It is capable of bi-directional flows of up to 0.9 $\mathrm {m/s}$ in its 2 $\textrm{m}$ long square test section with a cross section of 0.45 x 0.45 $\mathrm {m^2}$. The test section has three glass walls for optical access and illumination, and one stainless steel wall through which probes and various other equipment can be inserted, which can be seen in Fig. 2a . The bottom settling chamber is equipped to facilitate bubble injection through various methods (see below). The mean flow is driven by a 17.6 kW elbow pump (Egger RPP 300) controlled by a variable frequency drive (Danfoss VLT 175 H 1238ST). The mean water velocity is measured by an electromagnetic flow meter of 300 mm inner diameter (sensor: Danfoss Magflo MAG3100, converter: Siemens Sitrans Magflo MAG6000). A programmable logic controller (Unitronics V200-18-E5B) provides the feedback control on the water flow velocity and the bubble injection rate.

The top of the tunnel is in contact with the ambient air and acts as an overflow. The top bend above the test section is fitted with 5 corner turning vanes to aid in maintaining the streamwise cross-sectional uniformity of the flow further down. The top settling chamber contains laminarization meshes that consist of stainless steel wire screen meshes and polycarbonate honeycomb plates, each of which is 100 mm thick with a cylindrical cell size of 8 mm diameter (PC$-$8.0-CL from Plascore). Specifically, the sequence is from top to bottom: one wire screen of 2.5 mm mesh size and 0.7 mm thick, followed 360 mm further away by a sandwich stack of two wire screens of 0.93 mm mesh size and 0.34 mm thick with in between a honeycomb plate, followed 360 mm away by another copy of such a sandwich stack.

The bottom settling chamber provides means to inject bubbles into the flow. Bubble injection has been performed in the Twente Water Tunnel using different methods. One method is the usage of porous ceramic plates (Calzavarini et al. 2008; Mercado et al. 2012; Mathai et al. 2016). Micrometer-sized bubbles at around 150 $\mathrm {\mu m}$ can be injected when using this method. Alternatively, capillary "islands" can be installed in the bottom section to inject bubbles in the millimetric size range (Alméras et al. 2017). There are nine capillary islands, which contain a combined total of 621 capillary tubes with an inner diameter of $0.12\,\textrm{mm}$. In both cases, regular pressurized air at 5 bar is fed into the bubble injectors with a controlled mass flow rate (Bronkhorst EL-Flow F-202AV-M10-ABD-55-V) of up to 100 standard liters per minute.

### Jet array

The jet array consists of an array of 112 jet nozzles laid out in a staggered grid just above the test section of the tunnel. Each nozzle can fire a powerful water jet, independent of each other and controlled by computer following a predetermined protocol.

**Fig. 3:**
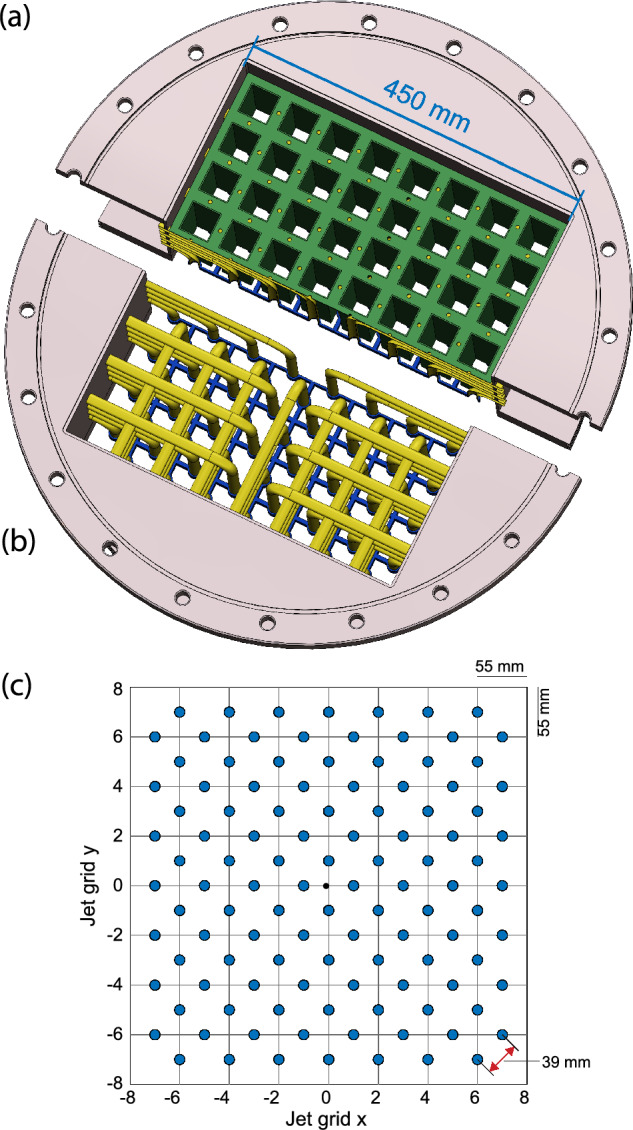
**a**, **b** A computer render of the jet array cut through into two halves for display purposes. Part (a) shows the array as installed inside the Twente Water Tunnel. The green part indicates an insert of square tubes that fits snugly from the top in between the jet pipes shown in yellow. Each square tube is internally 37 mm by 37 mm by 144 mm long and fully covers up the pipes. Part **b** has the square tubes removed to better show the arrangement of pipes. Each pipe is fed through the tunnel wall, bends down and ends in an exchangeable nozzle (not shown) held securely in place by the metal lattice, here colored in blue. **c** Coordinate system of the 112 jet nozzles laid out in a staggered grid. The light gray lines indicate the metal support lattice with a grid spacing of $55\,\textrm{mm}$ (indicated in the top right corner), each blue circle is a jet exit nozzle and the red arrow in the bottom left corner indicates the jet nozzle separation distance of $M=39\,\textrm{mm}$

**Fig. 4:**
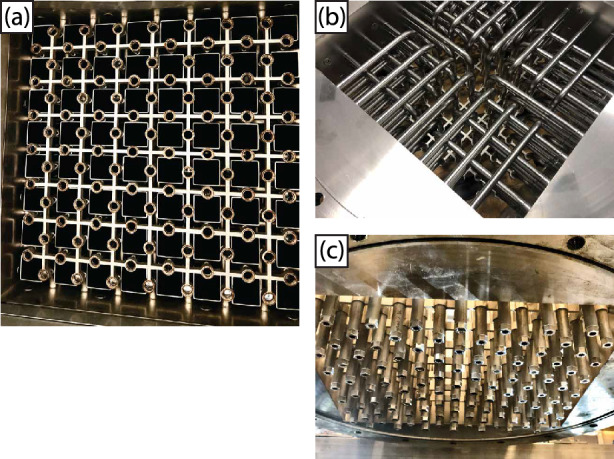
**a** The jet array inside the tunnel, without the nozzles installed, with the square tubes clearly visible. **b** A topview of the jet array outside the tunnel, where the tubes can be seen coming from the sidewall and going into the 3D-printed lattice. **c** A bottom view of the jet array outside the tunnel, with the nozzles installed

#### Mechanical construction

**Fig. 5:**
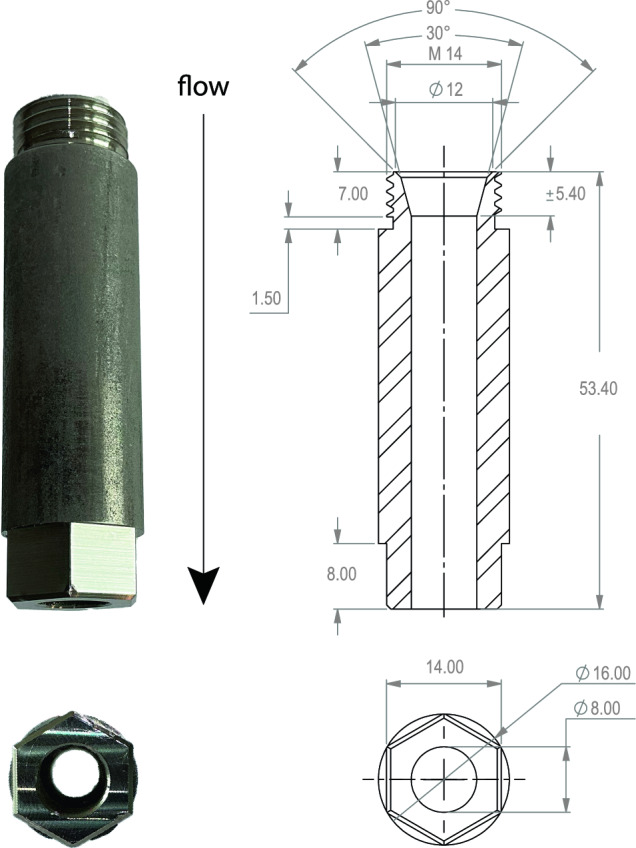
A schematic representation of the nozzle next to the image of an actual nozzle. All indicated dimensions are in millimeters

Figures 3 and 4 show the design of the jet array in detail. In total, 112 stainless steel (AISI 316) pipes of 12 mm inner diameter are fed through the tunnel wall, 28 pipes per side wall, and each pipe bends downwards to align streamwise with the mean "background" flow inside the test section of the tunnel (see Fig. 4b). Each pipe end is held in place by a stainless steel 3D-printed lattice with an array spacing of $55\,\textrm{mm}$ (see the blue colored part of panels (a) and (b) of Figures 3 and 4b), following a staggered grid layout with a jet separation distance of $M=39\,\textrm{mm}$ as shown in panel (c). Each pipe fits into an Orifice in the top half of the 3D-printed lattice. Each pipe has been vacuum brazed to the tunnel wall and the lattice using nickel as the soldering material. This was done to make the array section of the tunnel water tight and to ensure that the pipes are rigidly fixed in position. The bottom half of the lattice has threaded orifices in line with the pipes, which are visible in Fig. 4a, that can receive exchangeable nozzles to be able to tune the jet characteristics. Different nozzle exit diameters and exit cone shapes are possible this way. For the measurements presented here, we use a nozzle diameter of $8\,\textrm{mm}$ (see Fig. 5 for the schematic).

An insert of square tubes is installed over the pipes in order to make the fluid paths through the jet array more uniform (see the green part of Fig. 3a, and the visible squares above the 3D-printed lattice in Fig. 4a). This is necessary because the number of horizontal pipes decreases from the sidewalls toward the center of the array. Unmitigated, this would lead to an uneven background flow distribution with higher velocities at the center. Each square tube, also made of stainless steel (AISI 316), is internally 37 mm by 37 mm and 144 mm long, fitting snugly in between the pipes.

Pressure to the jet nozzles is provided by a pump, shown in Fig. 2b, which we call the "jet pump" to distinguish it from the main pump driving the mean background flow in the tunnel. It is a marine-grade 6.8-kW impeller pump capable of delivering 60 $\mathrm {m^3/h}$ at up to 5.2 bar driving pressure (Xylem Lowara 46SVH2N075T/4), powered by a 7.5 kW synchronous motor (Lowara PLM 132 B5) and controlled by a frequency inverter (Xylem Hydrovar 4.075). The pump can be driven at either a constant exit pressure Or at a constant shaft rotation rate. We have found that running the pump at a constant rotation rate provides the most stable operation, because pressure fluctuations induced by switching jets on and off further downstream can disturb the feedback control inside the pump controller when set to constant pressure mode. Running at a constant rotation rate is not a problem as we independently monitor the jet pressure in the manifolds. The allowable range of rotation rates for the pump is between 20 and $50\,\textrm{Hz}$.

**Fig. 6:**
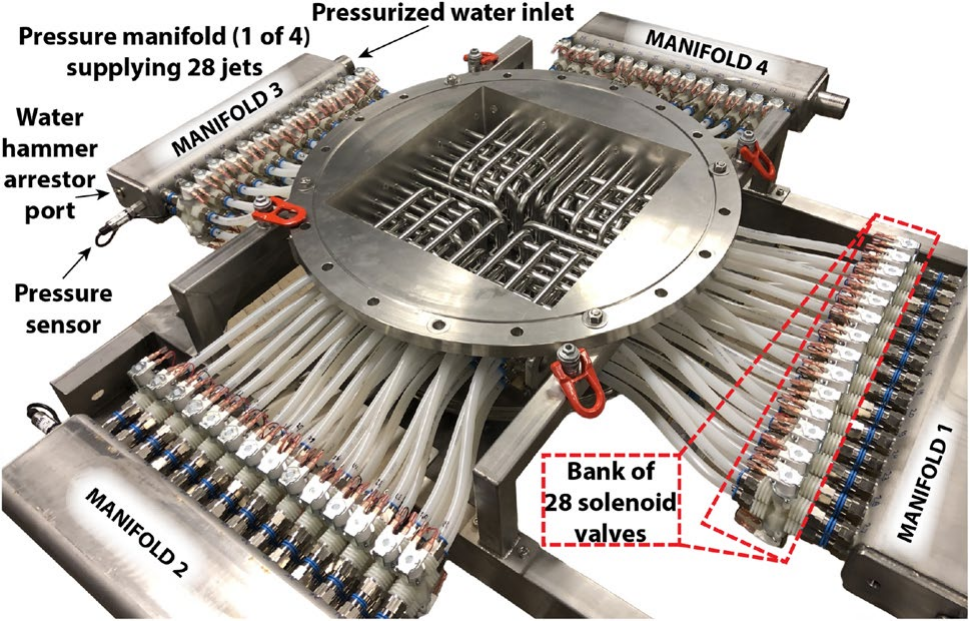
A photograph of the jet array before installation in the tunnel and without the square tubes in between the pipes. Each of the four manifolds is fed with pressurized water coming from the jet pump supplying pressure to 28 jet nozzles via independently controlled solenoid valves, resulting in 112 jets in total. The pressure of each manifold is monitored with a digital pressure sensor. A water hammer arrestor is mounted on each manifold (not present on the photo) to reduce potentially harmful pressure shocks induced by closing solenoid valves

The jet pump is fed with water coming from the bottom section of the tunnel’s recirculation (see Fig. 1). It bypasses the electromagnetic flow meter of the main tunnel flow. A single pipe takes the pressurized water up toward the jet array and is split by a hub into four separate flexible braided hoses, each connecting to one of four stainless steel pressure manifolds. Figure 6 shows a photo of the pressure manifolds and the jet array.

Each of the four pressure manifolds distributes pressure over 28 jet nozzles. A pressure sensor (Omega PXM309 007GI) with additional pressure snubber (Omega PS 4E MG) on the opposite end of the manifold to the inlet monitors the pressure in each of the manifolds and gets logged over time to a computer. A water hammer arrestor (Reliance Water Controls XVES 050 000) is installed adjacent to the pressure sensor on each manifold to reduce potentially harmful pressure shocks induced by switching jets on and off.

Solenoid valves sit in between the pressure manifolds and each of the 112 jet nozzles. The valves are closed unless activated, have a nylon body and a 11 mm diameter bore (RPE 5105NC-24VDC). All solenoid valves are individually controlled via a single microcontroller unit to be described in more detail in the following section. We measured a typical switching time from either fully closed to fully open, Or vice versa, of around 250 ms for these valves.

#### Electrical design

A single programmable microcontroller unit (MCU) is in control of individually opening and closing each of the 112 solenoid valves over time, following a predetermined and user-configurable protocol that is uploaded to the MCU in advance. The MCU is an Adafruit Feather M4 Express featuring an Atmel SAMD51 chipset running at a 120 MHz clock with 192 KB of RAM. The microcontroller can hold up to 5000 "frames" inside its memory where each frame encodes a particular on/off arrangement of all valves, making up a single protocol. The inter-frame time is 0.05 s long, meaning that the protocol will run for 250 s before repeating itself. We make use of an I/O expander board that provides 64 digital I/O channels connected over the I2C bus of the MCU. We combine two of these boards (Centipede Shield V2 by Macetech) to provide us with a total of 128 channels. We make use of 8 MOSFET boards (16 channel PLC isolation MOSFET board by Chifun K Workshop) to act as digital solid-state switches capable of driving, in our case, 14 solenoid valves each. These boards also galvanically isolate each input channel from their equivalent high-power output channel using optocouplers, which is important to protect the sensitive microcontroller from high-voltage spikes and ground noise. Serving both as a helpful troubleshooting tool and a flashy gimmick, a 16x16 RGB-LED matrix displays the current on/off status of each valve, according to the coordinate system shown in Fig. 3c. Additionally, the MCU also reads out the four pressure sensors at each of the pressure manifolds. The entire 3.3 V logic, including the MCU, is fully isolated from the high-voltage side and is powered by a separate floating power supply. The microcontroller unit, in turn, is connected via a USB isolator (Olimex USB-ISO) to a PC running a Python graphical user interface. From here new protocols can be generated, stored and uploaded into the microcontroller. The Python interface offers the user control of the jet pump and the jet array, plots the pressure of each pressure manifold and the pump in real-time and logs these pressures and the current "play" position of the protocol to a file on disk.

Additional redundancy and safety features have to be considered due to working with water pressures possibly in excess of 5 bars. Hence, a separate safety microcontroller operates besides the main microcontroller, shutting down and preventing the jet pump from turning on as soon as (1) all valves are closed or (2) if the safety microcontroller fails to receive an okay signal from the main controller once every 100 ms to prevent a potentially destructive pressure shock that could damage the system.

Details on the full electronic design and the source code of the microcontrollers and the graphical user interface can be found on the GitHub repository (van Gils 2024a).

## Jet array operating protocol

This section will describe how we orchestrate all jets of the jet array over both space and time. A *protocol* entails a set of open and close instructions over time for each of the solenoid valves. We base these instructions on a special kind of noise that allows us to straightforwardly control both the spatial and temporal coherence in the driving of the jet array and, with it, indirect control over the characteristics of the flow downstream of the array.

### OpenSimplex noise

Our implementation relies on OpenSimplex noise, which is a type of pseudo-random isotropic gradient noise that features smooth and coherent features in up to 4 dimensions. Gradient noise is generated by using a regular N-dimensional lattice, called a simplex, where random gradients are assigned to its vertices and then interpolated to create a smooth, continuous noise field. Here, isotropic means that the variance in the noise values along each dimension is equal. OpenSimplex noise was developed in 2014 (Spencer 2014) as an open source improvement of Simplex noise (Perlin 2001), which itself is based on Perlin noise (Perlin 1985).

**Fig. 7:**
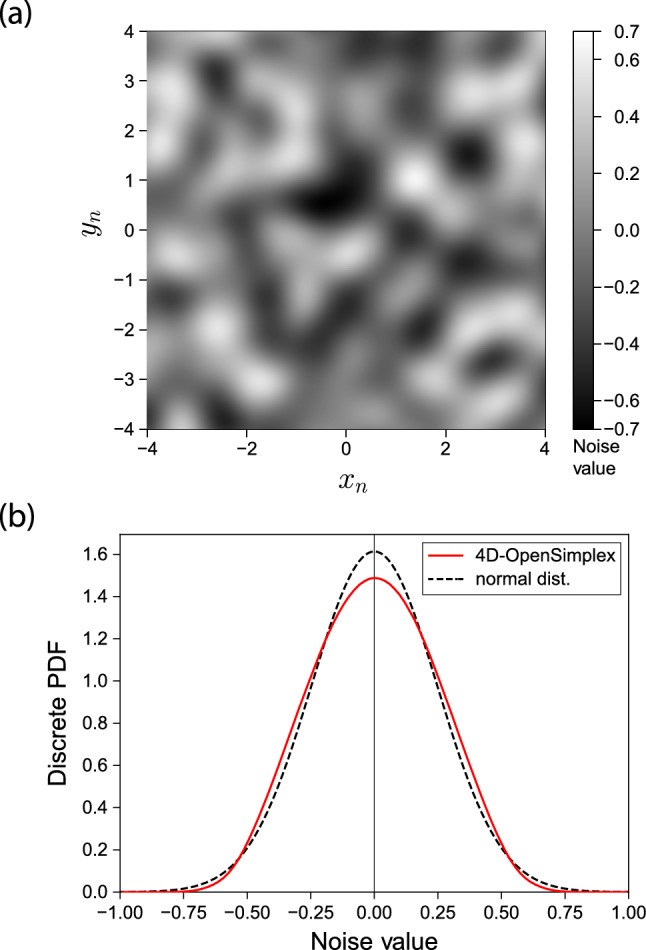
**a** A 2D slice out of 4D OpenSimplex noise in the plane $(x_n, y_n, z_n=0, w_n=0)$. The grayscale intensities correspond to the noise values, clipped in this particular image to the range $-$0.7 (full black) to 0.7 (full white) to increase the contrast for ease of visualization. No scaling has been applied to either the coordinates or the noise values. One can clearly observe the smooth and coherent nature of this type of gradient noise. **b** The discrete probability density function (PDF) of a large sample size of 4D OpenSimplex noise, shown in red. We sampled 100 × 100 × 100 × 100 randomly distributed points over the domains $[-100, 100]$ along each axis of the 4D space. For comparison we also show a normal distribution with an equivalent standard deviation, shown as the dashed black curve

In Fig. 7a, we show a two-dimensional slice taken out of four-dimensional OpenSimplex noise, represented here as a grayscale intensity raster image in Order to give the reader a feel for the smooth and coherent nature of this type of noise. We chose to present the 2D plane $(x_n, y_n, 0, 0)$, but any of the 2D planes out of the $(x_n, y_n, z_n, w_n)$-space will exhibit similar smoothness and coherence. Here, $x_n$, $y_n$, $z_n$ and $w_n$ lie along each of the four Orthogonal unit vectors of the 4D space in which the noise is defined with subscript *n* for "noise" used to distinguish these coordinates from the physical ones. The noise values are guaranteed to be within the range [− 1, 1], but the true extrema encountered over a certain sample region in $(x_n, y_n, z_n, w_n)$-space are not known a priori. Hence, we show in Fig. 7b the discrete probability density function (PDF) of the noise values taken out of a large sample size of 4D OpenSimplex noise (see the red curve). We sampled 100 × 100 × 100 × 100 randomly distributed points over the domains $[-100, 100]$ along each axis of the 4D space, resulting in a standard deviation of $\approx 0.247$ around zero mean. The dashed black curve shows a normal distribution with an equivalent standard deviation for comparison.

### 4D noise loop

The beauty of having gradient noise along four dimensions is that it allows us to navigate through this $(x_n, y_n, z_n, w_n)$-space in special ways. In particular for our use case the first two OpenSimplex dimensions $(x_n, y_n)$ are used to describe a rectangle that gets projected onto a 2D array, i.e., a grayscale raster image, which we will call a *noise frame*. This frame will have the OpenSimplex noise values evaluated at $(x_n, y_n, z_n, w_n)$ mapped to its $(x_{px}, y_{px})$-pixel locations as grayscale values.

The last two dimensions $(z_n, w_n)$ are used to describe a polar loop representing circular time as $(z_n=r \cdot \textrm{sin}(t), w_n=r \cdot \textrm{cos}{(t)})$, where *t* is the time and *r* is the radius of the time circle. We divide the time circle into equal segments and obtain a new frame for each new time.

**Fig. 8:**
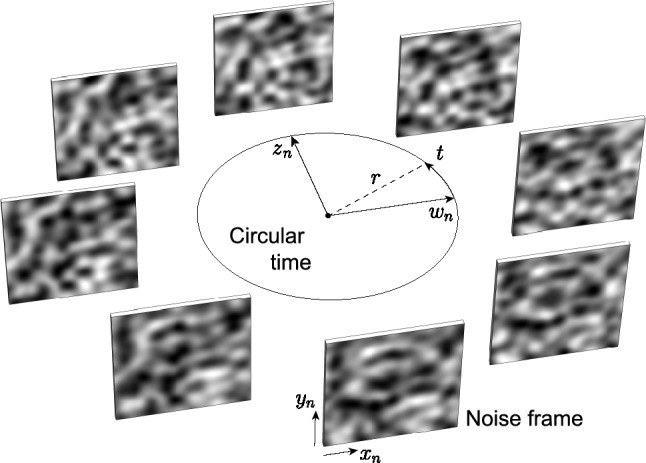
Demonstration of a four-dimensional OpenSimplex noise loop. The (*x*, *y*)-dimensions are used to describe a rectangle that is mapped onto a grayscale raster image called a *frame*. The (*z*, *w*)-dimensions are used to describe a circle in time along which new frames are evaluated. The collection of all frames is called a *stack*. The grayscale intensities of each frame are sampled from their respective (*x*, *y*, *z*, *w*) OpenSimplex noise values. Note the smooth and coherent nature of the noise inside of each frame *and* from frame to frame, seamlessly looping over time

This collection of frames we call a *noise stack*. The grayscale values in each frame correspond to the 4D OpenSimplex noise values evaluated at $(x_n, y_n, z_n, w_n)$ with each frame traversing through time in such a way that it seamlessly loops over and over again and with all frames fully unique from one to another. This is in essence a so-called 4D noise loop (see Figure 8 for a schematic representation).

We can use the smooth coherent nature of OpenSimplex noise to our advantage when constructing driving protocols for the jet array. Concretely, we generate a stack of $N_{frames}$ number of frames, each frame a grayscale raster image of $N_{px,x}$ pixels wide and $N_{px,y}$ high. A single $x_{px}$ or $y_{px}$-pixel increment inside this image will step by a factor of $X_{step}$ through the equivalent $(x_n, y_n)$ 4D OpenSimplex noise-space. We use the same $X_{step}$ factor for both the $x_{px}$ and $y_{px}$-directions to keep the mapping square. The radius *r* of the time circle is scaled linearly with $T_{step}$, considering $N_{frames}$ to be a constant. This means that a larger $T_{step}$ results in a larger temporal $(z_n, w_n)$-distance in between consecutive frames. Tying everything together gives:

1 $$\begin{aligned} x_n =\;&i_{px} \cdot X_{step},\end{aligned}$$

2 $$\begin{aligned} y_n =\;&i_{py} \cdot X_{step},\end{aligned}$$

3 $$\begin{aligned} z_n =\;&r \cdot \textrm{sin}(t),\end{aligned}$$

4 $$\begin{aligned} w_n =\;&r \cdot \textrm{cos}(t), \end{aligned}$$

with,

5 $$\begin{aligned} r =\;&\frac{N_{frames}}{2 \pi }\cdot T_{step},\end{aligned}$$

6 $$\begin{aligned} t =\;&2 \pi \cdot \frac{i_{frame}}{N_{frames}}, \end{aligned}$$

where $i_{px}$ denotes the x-pixel index running from 0 to $N_{px,x} - 1$, $i_{py}$ denotes the y-pixel index running from 0 to $N_{px,y} - 1$, and $i_{frame}$ denotes the frame index running from 0 to $N_{frames} - 1$.

Lastly, we will introduce the concept of a *feature size*, which is more intuitive to work with compared to using a step size. Simply put:

7 $$\begin{aligned} SFS =\;&1/X_{step},\end{aligned}$$

8 $$\begin{aligned} TFS =\;&1/T_{step}, \end{aligned}$$

where *SFS* and *TFS* are, respectively, the *spatial feature size* and *temporal feature size* parameters. Larger *SFS* means larger coherent structures apparent in single frames, and vice versa. Larger *TFS* means a single spatial structure remains coherent for a longer time duration in between frames, and vice versa. Note that $X_{step}$, $T_{step}$, *SFS* and *TFS* are all in noise-space units here. In Appendix A we show how we approximate *SFS* and *TFS* into physical units.

In summary: The parameters *SFS* and *TFS* allow for tuning of, respectively, the spatial and temporal coherent scales of the generated noise stack that will loop seamlessly over time. This forms the basis behind the driving protocols of the jet array. We have made our Python code for generating these and other four-dimensional OpenSimplex noise loops publicly available (van Gils 2024b) as a module with a well-documented application programming interface.

### Mixing scales

**Fig. 9:**
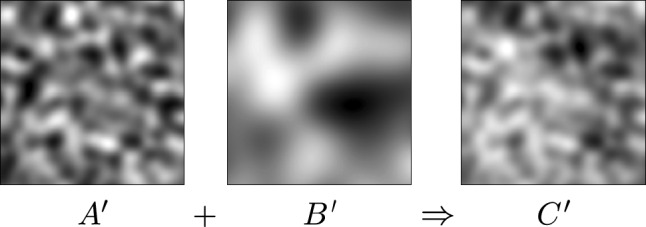
Mixing multiple scales together by blending. We show the first frames of noise stacks *A*, *B* and *C* which we each show inverted in their grayscale values for displaying purposes. Stack *C* contains a linear blend of the two distinct spatial feature sizes contained in stacks *A* and *B*

Instead of using a noise stack with a single set of feature sizes *SFS* and *TFS*, one could simply blend multiple noise stacks built with differing feature sizes together frame by frame to achieve multiple scales. This is depicted in Fig. 9. Assume we have a noise stack *A* built with a small $SFS_A$, and a noise stack *B* built with a larger $SFS_B$. Blending these stacks frame-by-frame together as $C = \frac{1}{2}A + \frac{1}{2}B$ results in stack *C*, now containing two distinct spatial feature sizes. The same can be done for the temporal feature size.

### Noise transformation

**Fig. 10:**
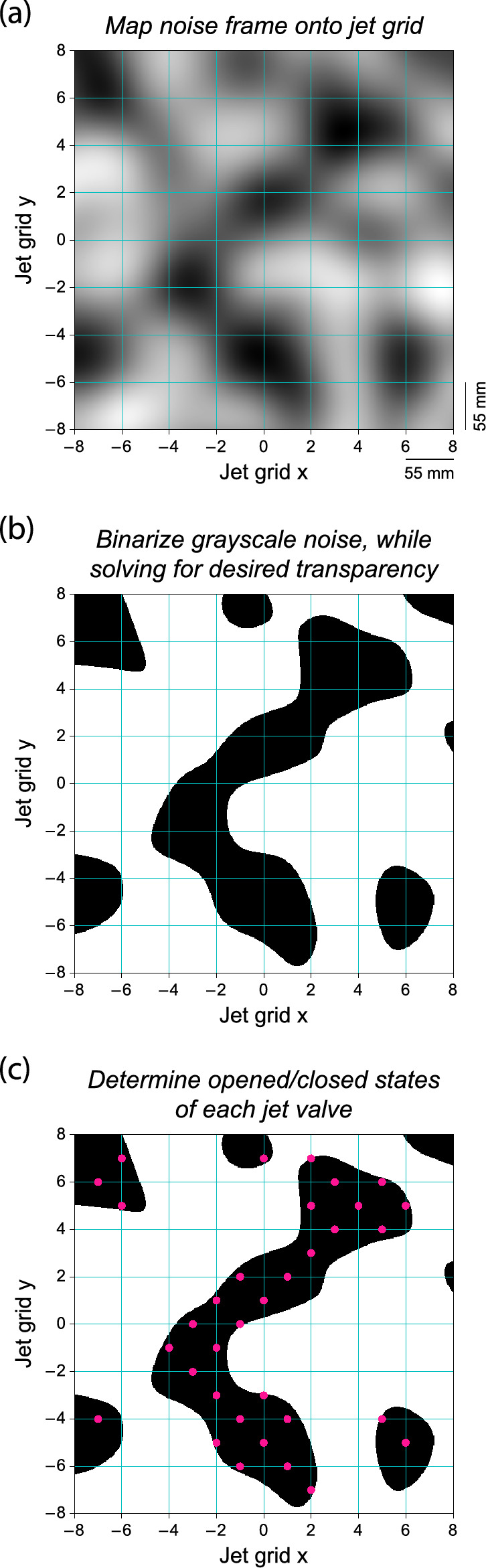
Steps toward transforming a noise frame (see panel (**a**)) into a frame decoding the states of each jet array valve (see panel (**c**)). Here, the target transparency to solve for was $\phi =0.3$, where transparency is defined as the fraction of "true" elements (black regions in panels (**b**) and (**c**)) with respect to all elements. The pink dots in (**c**) indicate opened valves. Closed valves are not shown

We need to transform the noise stack that contains frames with noise values inherently between -1 and 1 into a set of frames containing the binary open-or-closed states of the solenoid valves. We will illustrate the necessary steps in this transformation for a single set of protocol settings as an example, matching one of the data points shown later on in Sect. 4. To be specific: A single-scale protocol with $SFS=100$ (86 mm), $TFS=10$ (0.5 s) and a transparency (fraction of active jets) $\phi =0.3$. First, we map each noise frame onto the jet array coordinate system by scaling linearly to fit the array bounds (see Fig. 10(a)). Next, we binarize the -1 to 1 noise values for each frame in the stack. We use a dynamic threshold value that adapts per frame, to prevent large and unwanted pressure fluctuations inside the jet array due to stochastic extremes in the noise.

We binarize each frame by solving for a desired fixed transparency level, where transparency is defined as the percentage of "true" elements with respect to all elements. We employ a Newton solver that converges onto the specified threshold value (see Fig. 10b). Finally, we can determine the opened/closed states of each valve by querying the binary values at the array index locations (see Fig. 10c). The *noise stack* is now transformed into a *valve stack*.

**Fig. 11:**
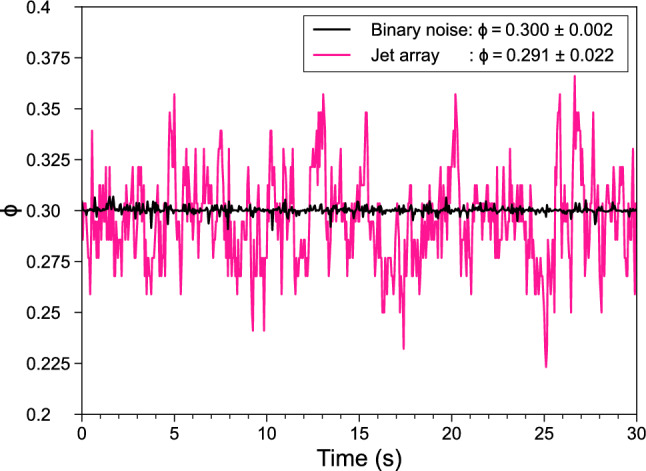
The transparency over time after binarizing the noise while solving for a transparency of $\phi =0.3$ (black curve) and after subsequently mapping it to discrete valve locations (pink curve). Only the first 30 s of the full 250 s ($N_{frames}=5000$, $\Delta t_{frame}=0.05$ s) are shown. The fluctuations in the jet array transparency are acceptable for our water pressure system

The resulting transparencies over time for this particular example are shown in Figure 11.

The transparency of the binarized noise (black curve) is converged within 0.002-point standard deviation. The transparency of the jet array (pink curve), defined as the percentage of opened valves over all valves, is fluctuating more: $2.2\%$-point. This is because of a coarsening effect when mapping onto a sparser set of valve locations. Regardless, fluctuations of this magnitude pose no practical problems and are acceptable for our water pressure system.

**Fig. 12:**
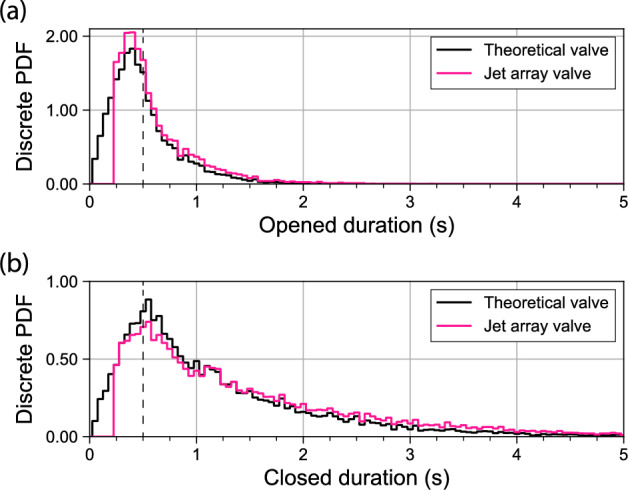
The ensemble statistics over all valves for the full 250 s of this particular protocol. The bin size is equal to the inter-frame time of 0.05 s. The black curves indicate the discrete PDFs taken directly from the unadjusted valve state time series. The pink curves indicate the PDFs after the smallest segments of both opened duration and closed duration have been removed from the time series, accounting for a minimum needed switching time of 0.25 s of our used jet array valves. Note that the peak in both distributions indeed matches the set temporal feature size of $TFS = 0.5$ s indicated by the dashed vertical lines

Out of the valve stack we construct the time series of the state per each solenoid valve. The ensemble statistics over all valves for the full 250 s of the protocol are shown in Figure 12, where we plot the discrete PDFs of the opened duration in panel **a**, and those of the closed duration in panel **b**. The black curves indicate the distribution when theoretical valves with infinitely fast switching times are considered. Note that the peak of both opened and closed distributions match with the chosen temporal feature size of $TFS = 0.5$ s as indicated by the dashed vertical lines.

### Finalizing the protocol

Before we send the valve stack as a finished protocol to the jet array microcontroller for playback, we make a final correction to try to mitigate the wear on our solenoid valves. We enforce a minimum state duration of 0.25 s, which coincides with the measured time for our valves to switch from completely closed to a full outflow. Any segments from the valve state time series that are smaller than the minimum switching time are iteratively replaced with their previous state, taking into account that the time series are looping end-to-start. This distribution of the adjusted durations is shown as the pink curves of Fig. 12. The redistribution of the removed small segments does not have a significant impact on the quality of the PDFs at larger durations. Likewise for the jet array transparency that is minutely affected by this correction. The caveat is that the *TFS* must remain larger than the minimum switching time of the valves. Finally, we transform all adjusted valve state time series back into a valve stack. It is then saved to disk and uploaded as a jet array protocol for playback by the microcontroller.

## Jet array characterization

Having covered the physical apparatus and operating protocols, we now turn to the flow conditions in the test section of the tunnel. We begin by examining the jet exit velocity and outline the measurement setup. Next, we discuss the effects of pump frequency and transparency (i.e., the fraction of active jets at a given time) on the flow, the impact of spatial feature size, the development of streamwise flow, and, finally, the homogeneity and isotropy of the flow.

### Jet exit velocity

**Fig. 13:**
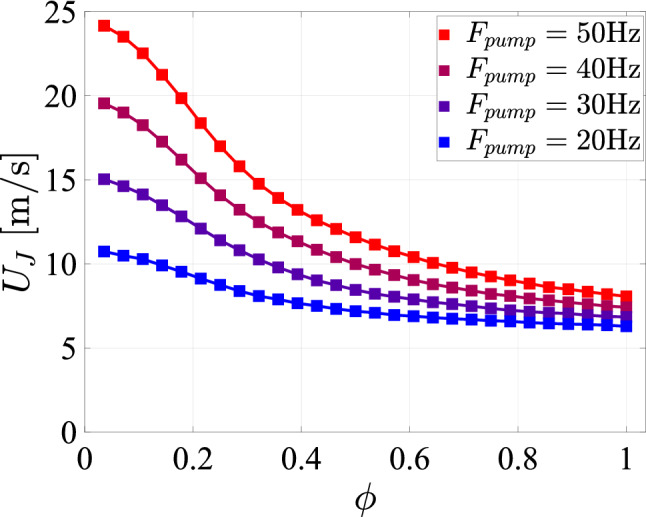
The estimated jet exit velocities plotted against the transparency corresponding to the measurements shown in Fig. 32

Although we are not able to directly measure the jet exit velocity, we can estimate its magnitude from the manifold pressures which we do monitor (see Fig. 6 for the location of the pressure sensors on the manifolds). To achieve this, we model the flow path between the manifold and the jet exit as a series of pipes and obstructions. This then allows us to estimate the jet exit velocity, $U_J$, for a given manifold pressure. Details of the model and the specific values used to determine the pressure drop can be found in Appendix B. The resulting jet velocity estimates for continuous operation (i.e., without switching valves) as a function of transparency and for different pump settings are shown in Fig. 13.

**Fig. 14:**
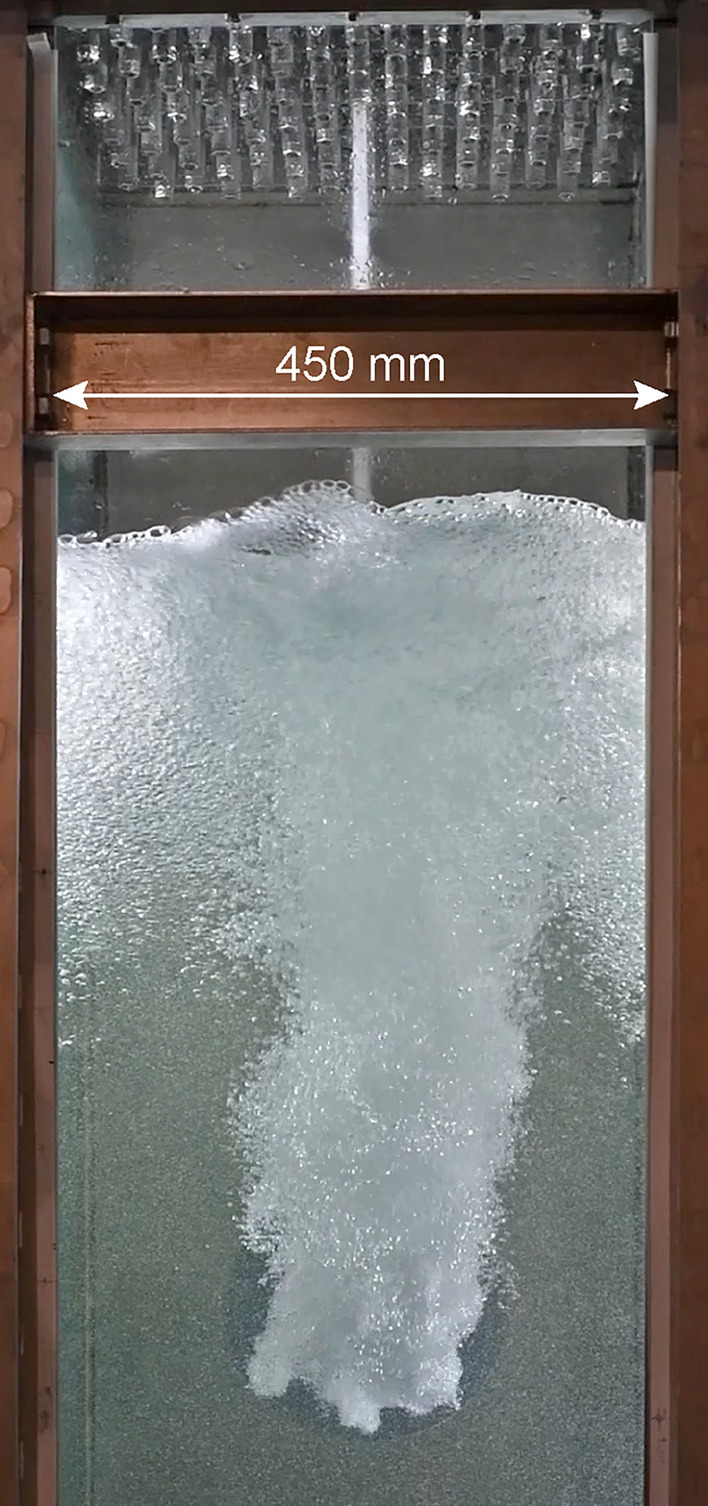
A single jet of the jet array hitting the free water surface with a driving pressure of 5 bar. The 112 jet nozzles are visible in the top of the image. For displaying purposes only do we jet onto a free water surface. Nominal operation would have the array completely submerged

The decrease of $U_J$ with increasing transparency corresponds to the drop in pump head at increased flow rate. Similarly, a reduction in the pump frequency, the rotation rate of the pump shaft, results in a lower pump head and hence lower jet velocities. According to our estimates, the jet exit velocity at the highest pump setting is still $\approx 8\;m/s$ at a transparency of $\phi =1$, i. e. if all valves are open. This value increases beyond 20 *m*/*s* if only a small fraction of the jets is firing at low transparency. To provide an impression of the array in action, Fig. 14 shows a single jet impinging onto a free water surface using the nozzle design of Fig. 5. Note that the water level is lowered here for illustration purposes only, nominal operation would have the array fully submerged.

**Fig. 15:**
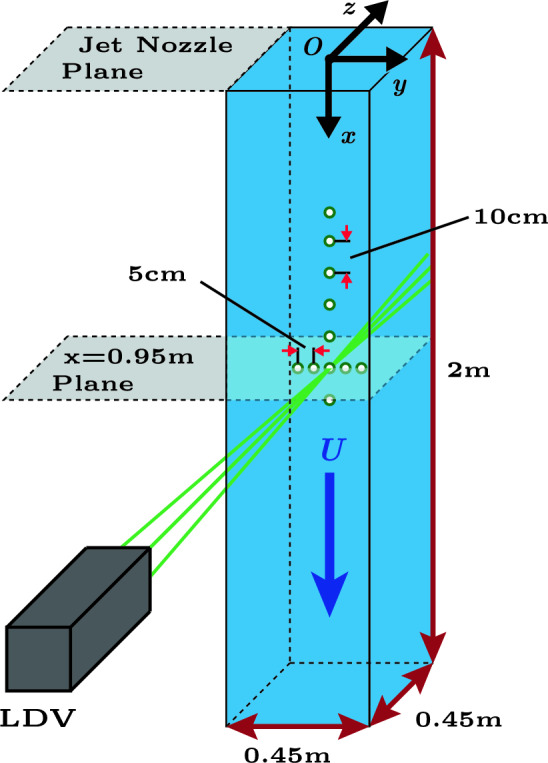
A diagram showing a representation of the test section, with the gnomon indicating the axes directionality, where the origin of the coordinate system is located on the centerline in jet array nozzle exit plane, and where *U* indicates the applied background velocity. The queried measurement locations are indicated (approximately to scale) using the green-edged dots

The total volumetric flow through the test section is the sum of the applied background flow *U*, determined by measuring the volumetric flow rate with the electromagnetic flow meter in the return section of the main flow loop (see Fig. 1), and the volume injected by the jet array. Therefore, the mean vertical velocity based on the volumetric flow rate ($U_x$) (see Fig. 15) is equal to the sum of *U* and the resultant velocity from the volume injected by the jets, spread over the test section cross-sectional area. We can therefore estimate the mean vertical velocity using the jet exit velocity estimates as

9 $$\begin{aligned} U_x=U + U_{TJ} \end{aligned}$$

where $U_{TJ}$ is the contribution to the mean vertical velocity resulting from the volume injected by the jets, Or, in other words, the tunnel velocity resulting from the jets. With a total number of 112 jets, the estimated average injected volume by all active jets is

10 $$\begin{aligned} U_{TJ} = 112\cdot \phi \cdot U_J\cdot A_J/A_T \end{aligned}$$

with $A_J$ and $A_T$ being the cross-sectional areas of a jet nozzle and of the test section, respectively. The estimated mean velocity is therefore given by

11 $$\begin{aligned} U_x = U + 112\cdot \phi \cdot U_J\cdot \frac{A_J}{A_T}. \end{aligned}$$

Note that $U_x$ is an upper estimate since it does not account for transients due to the switching of the valves. Various figures in subsequent sections will show a black dashed line to indicate the mean velocity estimate where it is important to emphasize that *U*, $U_x$, and $U_{TJ}$ are all spatial averages, while the measurement shown in those figures are point measurements.

### Flow characterization

As is clear from the previous sections, there are a large number of tunable parameters that can affect the flow conditions. These system parameters include:

- The background flow velocity
- The jet pump frequency
- The transparency (the fraction of active jets)
- The protocol spatial feature size
- The protocol temporal feature size

Exploring the full parameter space would require a time-consuming, extensive and expensive measurement campaign. Therefore we will limit our effort to exploring the effect that changing a limited number of these parameters has on the flow, while keeping the others constant. The background velocity was set to $20\;\mathrm {cm/s}$, which is near the rise velocity of millimetric bubbles (David et al. 1953). Additionally, we kept the protocol temporal feature size fixed at $TFS=0.5\,\textrm{s}$ and limited the number of scales in each protocol to one for the protocols we generated.

#### Measurement setup

The flow characterization measurements are performed using a laser Doppler velocimetry system (Dantec Dynamics FlowExplorer). We use polyamide particles with a diameter of $5\,\mathrm {\mu m}$ (Polyamide Seeding Particles, PSP-5 from Dantec Dynamics) as tracers. The LDV is mounted on a traverse, which allows us to easily move the LDV to measure at various locations in the test section of the tunnel. At each location, both the longitudinal (*x*-component, $u_x$) (see Fig. 15) and the transverse (*y*-component, $u_y$) velocities were measured for 15 min. All queried measurement locations are shown in Figure 15 and are at $x>>M$.

As the signal provided by the LDV system relies on the passage of particles in the sampling volume, additional steps must be taken in the processing of the acquired data to correct for sampling bias. For the determination of the mean and standard deviations of the measured velocity components, we use transit time weighing (Damaschke et al. 2018),

12 $$\begin{aligned} \langle u_i\rangle =\;&\dfrac{\sum _{\alpha =1}^{\mathcal {N}}u_{i,\alpha }\tau _{i,\alpha }}{\sum _{\alpha =1}^{\mathcal {N}}\tau _{\alpha }}\end{aligned}$$

13 $$\begin{aligned} \langle u_i'\rangle _{\textrm{RMS}}^2=\;&\dfrac{\sum _{\alpha =1}^{\mathcal {N}}\tau _{i,\alpha }(u_{i,\alpha }-\langle u_i\rangle )^2}{\sum _{\alpha =1}^{\mathcal {N}}\tau _{i,\alpha }} \end{aligned}$$

where $u_{i,\alpha }$ is the discrete velocity component in the *i* direction measured at particle arrival time $t_{i,\alpha }$, with $i\in [x,y]$ and $\mathcal {N}$ the total number of data points in a measurement, $u_i'$ is the fluctuating velocity component in the *i* direction, obtained via a Reynolds decomposition $u_i'=u_i-\langle u_i\rangle$. Further, $\tau _{i,\alpha }$ is the particle transit time, $\langle u_i\rangle$ is the weighted mean velocity, and $\langle u_i'\rangle _{\textrm{RMS}}^2$ is the weighted velocity variance. Similarly, the skewness of a velocity component is calculated as

14 $$\begin{aligned} \gamma _{i} = \dfrac{\sum _{\alpha =1}^{\mathcal {N}}\tau _{i,\alpha }(u_{i,\alpha }-\langle u_i\rangle )^3}{\langle u_i'\rangle _{\textrm{RMS}}^3\sum _{\alpha =1}^{\mathcal {N}}\tau _{i,\alpha }} \end{aligned}$$

We calculate the velocity probability density functions by using the particle transit times as binning weights. Therefore the value of the discrete velocity PDF of $u_i$ is calculated as

15 $$\begin{aligned} PDF(\mathcal {U}_{i,\alpha }) =\;&\dfrac{b_\alpha }{\sum _{\alpha =1}^{N}b_\alpha } \end{aligned}$$

16 $$\begin{aligned} \begin{aligned} b_\alpha (\mathcal {U}_{i,\alpha }) =\;&\sum _{\beta =1}^{{N}}\tau _{i,\beta }\textrm{H}\left( u_{i,\beta }-\left( \alpha -\dfrac{1}{2}\right) \cdot \Delta \mathcal {U}_i\right) \cdot \\&\left( 1-\textrm{H}\left( u_{i,\beta }-\left( \alpha +\dfrac{1}{2}\right) \cdot \Delta \mathcal {U}_i\right) \right) \end{aligned} \end{aligned}$$

where $PDF(\mathcal {U}_{i,\alpha })$ is the value of the discrete PDF of the velocity in the *i* direction in a bin with central velocity $\mathcal {U}_{i,\alpha }$. The bin width is $\Delta \mathcal {U}_i$, *N* is the number of bins, $b_\alpha$ is the bin weight, and $\textrm{H}$ is the Heaviside step function.

For the calculation of the second-order structure functions,

17 $$\begin{aligned} D_{ii}(r) = \langle \left( u_i(x+r,t)-u_i(x,t)\right) ^2\rangle \end{aligned}$$

where *r* is the spatial separation of measurement points, the temporal LDV data must be converted to a spatial dataset. Since the turbulence intensity ($u'/\langle u_x\rangle$) in our system is higher than 10% (see Figs. 22 and 27), the validity of using Taylor’s frozen turbulence hypothesis for converting the temporal data to the spatial domain is questionable. We therefore use the "convection record" method developed by Buchhave and Velte (2017) to obtain the spatial displacement $s_{\alpha }$ as

18 $$\begin{aligned} s_{\alpha } = \sum _{n=1}^{\alpha }|\vec {u_{n}}|\Delta t_{i,n} \end{aligned}$$

with $|\vec {u_{n}}|$ being the instantaneous velocity magnitude and $\Delta t_{i,n}$ the sample inter-arrival time. The instantaneous velocity magnitude is estimated by dividing the diameter of the LDV measurement volume $D_{LDV}=0.1\textrm{mm}$ by the transit time,

19 $$\begin{aligned} |\vec {u_n}|\approx \frac{D_{LDV}}{\tau _{i,n}}. \end{aligned}$$

**Fig. 16:**
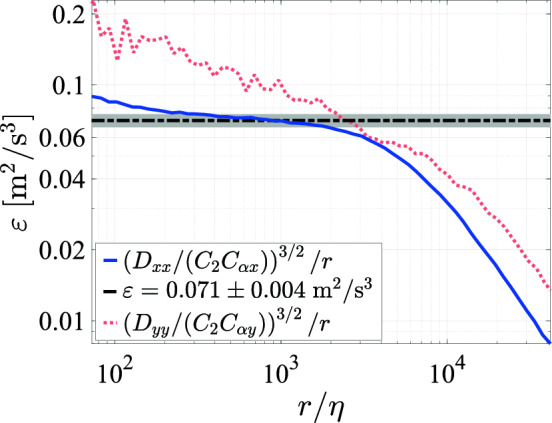
Compensated structure functions for the *x* (blue) and the *y* (red dotted) velocity components using $C_2=2.0$, $C_{\alpha x}\approx 1.21$, and $C_{\alpha y}\approx 1.24$, with the gray dashed line indicating the fit to obtain the dissipation rate, and the shaded area indicating the error

We then create an array of spatially uniformly distributed samples, with steps of $\Delta s$ being the smallest calculated displacement. The values of the velocity array are

20 $$\begin{aligned} v_{i,\kappa } =v_{i}(\kappa \Delta s)= {\left\{ \begin{array}{ll} u_{i,\alpha }\quad & \textrm{if}\,|s_{\alpha }-\kappa \Delta s|<\Delta s/2\\ 0& \textrm{if}\,|s_{\alpha }-\kappa \Delta s|\ge \Delta s/2 \end{array}\right. } \end{aligned}$$

and the values of the weights array are

21 $$\begin{aligned} w_{i,\kappa } = {\left\{ \begin{array}{ll} \tau _{i,\alpha }\quad & \textrm{if}\,|s_{\alpha }-\kappa \Delta s|<\Delta s/2\\ 0& \textrm{if}\,|s_{\alpha }-\kappa \Delta s|\ge \Delta s/2 \end{array}\right. } \end{aligned}$$

where $\kappa$ is an integer in the range $[1,\mathcal {M}]$ with $\mathcal {M}=s_{\mathcal {N}}/\Delta s$. The structure function is then calculated as

22 $$\begin{aligned} D_{ii}(r_\zeta )=\dfrac{\sum _{\xi =0}^{\mathcal {N}-\zeta -1}(v_{i,\xi +\zeta }-v_{i,\xi })^2w_{i,\xi +\zeta }w_{i,\xi }}{\sum _{\xi =0}^{\mathcal {N}-\zeta -1}w_{i,\xi +\zeta }w_{i,\xi }} \end{aligned}$$

where

23 $$\begin{aligned} r_\zeta =\zeta \Delta s, \end{aligned}$$

where $\zeta$ is an integer in the range $[1,\mathcal {M}]$ and $\zeta \Delta s$ is the spatial shift used for calculating the structure function from the resampled velocity data. We determine the energy dissipation rate $\varepsilon$ by fitting a horizontal line to the plateau region of the structure functions compensated with the theoretical scaling for the inertial sub range given by

24 $$\begin{aligned} D_{ii}=C_{\alpha i}C_{2}(\varepsilon r)^{2/3} \end{aligned}$$

for the structure function of the velocity component $u_i$, where $C_2=2.0$ is a universal constant (Pope 2000). Due to the application of the convection record method, the displacement is not strictly in any singular direction. Therefore $D_{xx}\ne D_{LL}$ and $D_{yy}\ne D_{NN}$ as would be the case under Taylor’s frozen turbulence hypothesis. The values of the coefficient $C_{\alpha ,i}$ are therefore not known apriori and must be determined based on the distribution of the convection velocities. The derivation of $C_{\alpha ,i}$ and the calculation of the values in our case are elaborated upon in Appendix C. We show an example of the compensated longitudinal and lateral second-order structure functions, from measurements at $0.95\textrm{m}$ below the array in the center of the tunnel, with the pump set to the maximum frequency of $50\,\textrm{Hz}$ and a transparency of $\phi =0.3$, in Fig. 16. We find an energy dissipation rate of $0.071\pm 0.004\,\mathrm {m^2/s^3}$ for the shown example, based on the longitudinal structure function. The value of $\varepsilon$ obtained in this way is generally found to be of the same order of magnitude as the transversal structure function. However, since the average sampling rate in the lateral direction was much lower (approximately $36\textrm{Hz}$, vs. approximately $797\textrm{Hz}$ in the longitudinal direction in the present example), the convergence of $D_{yy}$ in the inertial range is insufficient for this case.

#### Flow dependence on pump frequency and transparency

From Fig. 13, it is clear that the effect of increasing the transparency is to lower the jet exit velocity, and that a decrease in pump frequency will do the same. To test how this affects flow parameters such as the turbulence intensity ($u'/\langle u_x\rangle$), we varied the transparency for a set pump frequency of $50\,\textrm{Hz}$ and a constant spatial feature size of $SFS=129\,\textrm{mm}$.

**Fig. 17:**
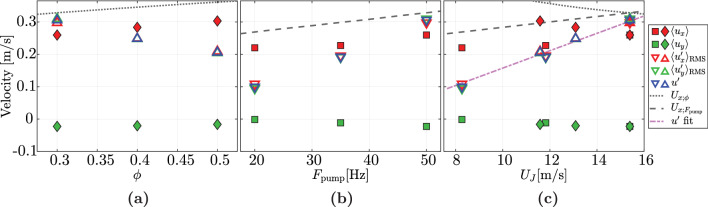
The mean, standard deviation and the velocity fluctuations of the lateral and longitudinal velocities plotted against: **a** the array transparency for a constant pump frequency of $50\,\textrm{Hz}$, **b** the pump frequency for a constant transparency of $\phi =0.3$, and **c** the jet exit velocity, where **c** contains the data from Figures **a** and **b** represented using the same symbols. The dotted line in Figure (**a**) and the dashed line in Figure (**b**) represent the $\langle u_x\rangle$ estimate corresponding to Eq. 11, and are also both included in Figure (**c**)

The results of corresponding measurements taken on the centerline of the tunnel $95\textrm{cm}$ below the array are shown in Fig. 17a. In general, the turbulence intensity is high since the velocity fluctuations ($\langle u_x'\rangle _{\textrm{RMS}}$, $\langle u_y'\rangle _{\textrm{RMS}}$, $u'=\sqrt{\langle u_x'\rangle _{\textrm{RMS}}^2+2\langle u_y'\rangle _{\textrm{RMS}}^2}/\sqrt{3}$) are comparable in magnitude to the mean flow velocity in the *x*-direction. Further, the mean velocity in the *y*-direction remains at least 5 times smaller than the mean velocity in the *x*-direction. We also observe that the velocity fluctuations in the *x*- and *y*-directions are approximately equal, indicating near-isotropic conditions. Figure 17a shows that an increase in the transparency leads to a decrease in the magnitudes of the velocity fluctuations. This result differs from the findings of Variano and Cowen (2008), who report an increasing magnitude of the velocity fluctuations with increasing $\phi$, up to a certain optimal value (at $\phi =0.125$ in their case), after which $u'$ declines with increasing $\phi$. However, in their experiments the jet exit velocity remained constant for varying $\phi$, whereas the pump frequency was not changed in our experiments. This results in a lower jet exit velocity for a higher transparency, which is consistent with the estimates shown in Fig. 13. Similar to the measurements where we change the transparency, we also performed several measurements at the same location in the tunnel, where we varied the pump frequency, but kept the transparency at $\phi =0.3$ and the spatial feature size at $SFS=129\,\textrm{mm}$. Figure 17(b) presents the results of these measurements, showing that an increase in pump frequency—and consequently of the jet exit velocity—leads to a rise in the magnitude of the velocity fluctuations.

We attribute both the increase of the velocity fluctuations with increasing pump frequency and for decreasing transparency to the resulting increase in $U_J$. This is confirmed by Fig. 17c, where we plot the same data directly vs. the estimate for $U_J$, revealing a clear and approximately linear proportionality between the jet velocity and the magnitude of the velocity fluctuations. This finding is in line with previous research, such as in Pratt et al. (2017); Masuk et al. (2019), and Ghazi Nezami and Johnson (2024), where an increase in the turbulent fluctuations is observed for an increase in jet exit velocity. In McCutchan and Johnson (2023) an increase in the jet exit velocity is also found to lead to an increase in the turbulence kinetic energy, however only for certain "optimal" $\phi$. Additionally, the magnitude of the velocity fluctuations is found to scale linearly with the jet exit velocity (Tan et al. 2023) according to

25 $$\begin{aligned} \frac{u'}{U_J}=\frac{C_0BD_J}{x}, \end{aligned}$$

where *B* is the velocity decay constant that depends on the velocity ratio between the jet exit velocity and the surrounding co-flow (Or et al. 2011), which is $U_J/U\approx 75$ in our case. The value of *B* for the closest velocity ratio reported in Or et al. (2011) ($U_J/U=20$) is 5.47, which is what we use (similar to Tan et al. (2023)). $C_0$ is a coefficient of proportionality, approximately equal to 0.28 (Pope 2000; Tan et al. 2023). We include a linear fit to the data measured at $x=0.95\,\textrm{m}$ in Figure 17(c). We note that the relation reported in Tan et al. (2023) (equation 25) does not fully conform to our measurements, unless an offset of $\beta _2 = -0.11\,\mathrm {m/s}$ is introduced such that $u' = \beta _1 U_J + \beta _2$, with $\beta _1 = 0.027$. Interestingly, the mean velocity either increases or decreases with increasing $U_J$, depending on whether the pump frequency or the transparency is varied. These trends are consistent between the data and the corresponding estimates of $\langle u_x \rangle$ based on equation (11), which are also included as dashed and dotted black lines in Figure 17 (c).

**Fig. 18:**
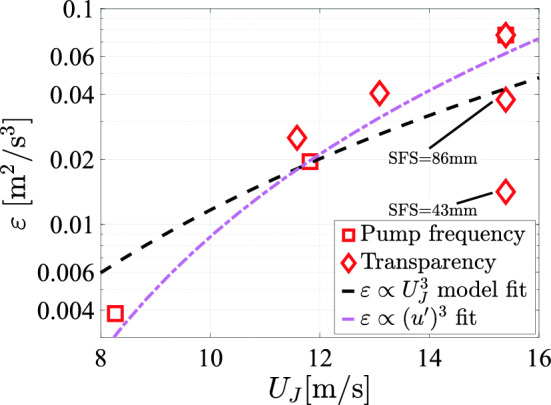
The energy dissipation rate plotted against the jet exit velocity, with the energy dissipation rate calculated from the data corresponding to the measurements of Figs. 17 and 19. In the latter, the protocol spatial feature size was varied. All measurements except for two, which are annotated with their respective spatial feature size, were performed with $\textrm{SFS}=129\,\textrm{mm}$. The black and magenta lines indicate a direct cubic fit, and a fit corresponding to the linear fit shown in Fig. 17c

This means that similar turbulence levels can be reached at different mean flow velocities by suitably varying transparency and pump power. Practically, this implies an additional way to decouple turbulence generation from the mean flow. As expected, $U_x$ is seen to overestimate the data, typically by about 30% (here and in subsequent plots), since it does not account for transient effects. Given the linear dependence of $u'$ on $U_J$, the turbulent energy dissipation rate $\varepsilon$ is expected to scale with $U_J^3$, as in Tan et al. (2023). We plot the measured energy dissipation rate against the jet exit velocity in Figure 18. The black dashed line indicates a fit assuming $u' \sim U_J$ without an offset correction, which does not conform well to the data. The agreement with the data is much better, when the offset correction $\beta _1$ is taken into account in the estimation of $u'$, which corresponds to the fit shown in Fig. 17c.

#### Protocol spatial feature size

Now that we have shown the effect that the jet exit velocity has on the flow statistics of the flow in our facility is, and that it is in line with previous findings, we turn to investigating the effect that the spatial feature size of the protocol has on the flow conditions. From previous work (Variano and Cowen 2008; Pérez-Alvarado et al. 2016; Carter et al. 2016), we know that the properties of the operating protocol, such as the mean and standard deviation of the jet on- and off-times, can have a strong effect on the flow characteristics. Additionally, Pérez-Alvarado et al. (2016) have shown that a spatially coherent distribution of active jets has a negative effect on the turbulence characteristics of the flow. However, in the protocols they tested, the distribution of active jets was always approximately homogeneous across the jet array, meaning that the protocols do not exhibit persistent “clusters” of jets. As has been explained in Sect. 3 and as can be seen in Fig. 9, increasing the spatial feature size in our protocols increases the dimensions of the regions of active jets. As a result, a protocol with a larger spatial feature size exhibits larger jet clusters as compared to a protocol with a smaller spatial feature size, without increasing the number of active jets. Not much is known about the effect that this spatial feature size has on the flow. This point is interesting, since unlike the other parameters considered so far, changing the feature size does not affect the number of active jets or the jet exit velocity.

**Fig. 19:**
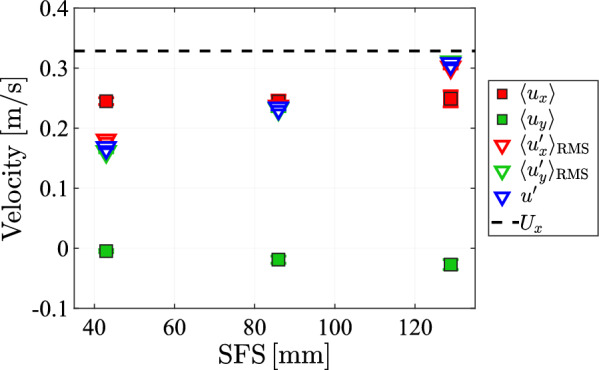
The mean, standard deviation and the velocity fluctuations of the lateral and longitudinal velocities plotted against the protocol spatial feature size

To investigate the effect that the protocol spatial feature size has on the flow, we measured the flow statistics on the centerline of the tunnel $95\,\textrm{cm}$ below the array, for 3 different feature sizes SFS at a pump frequency of $50\,\textrm{Hz}$ and with a transparency of $\phi =0.3$. The result of these measurements are shown in Figure 19. There is a significant increase in the fluctuations of both the lateral and longitudinal velocity components, indicating stronger turbulence at larger spatial feature size. This does not affect isotropy, as fluctuations of both components remain of approximately the same magnitude. Also, the vertical mean velocity remains unchanged across all SFS settings, while the mean in the lateral component decreases slightly but remains small compared to the fluctuations.

Since the velocity fluctuations strongly depend on the protocol scales, we want to know if this also holds for the energy dissipation rate. In Fig. 18 we see that the energy dissipation rate also strongly depends on the protocol spatial feature size. Thus varying the protocol spatial feature size is an effective method of changing turbulence characteristics without affecting the mean flow velocity or the jet exit velocity. It is noteworthy that these results differs from the ones in Tan et al. (2023), who found the dissipation rate to be largely independent of the array spacing for a configuration in which all jets were firing continuously. The difference may be related to the interaction (Lin and Sheu 1991; Tanaka 1974) of closely spaced jets in our protocols.

**Fig. 20:**
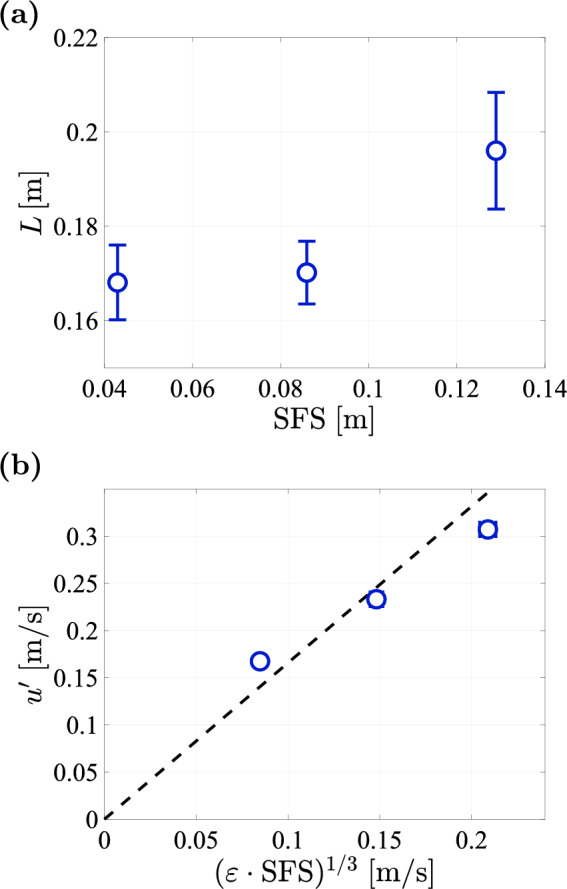
**a** Integral length scale plotted against the spatial feature size **b** Magnitude of the velocity fluctuations plotted against the product of $\textrm{SFS}$ and $\varepsilon$, with the black dashed Line representing a Linear fit with slope 1. For both plots $\phi =0.3$ at a pump frequency of $50\,\textrm{Hz}$

In order to assess the influence of the spatial feature size on the large scale turbulence, we plot the integral length scale *L* against the spatial feature size in Figure 20 (a). Here, *L* is determined by fitting an exponential function of the form $f(r)=\exp {(-r/L)}$ to the autocorrelation function (Johnson and Cowen 2018; Bang and Pujara 2023; Ghazi Nezami and Johnson 2024). As Fig. 20a shows that increasing the spatial feature is associated with larger values for the integral length scale. We note that since the integral length scale and the tunnel cross-sectional dimensions are of a similar order of magnitude, the integral length scale estimation may lack accuracy.

However, this trend is not linear, such that the magnitude of the velocity fluctuations does not exactly follow the scaling $u'\sim (\varepsilon \cdot \textrm{SFS})^{1/3}$, which would be expected for $L \sim SFS$ (see Fig. 20b).

#### Streamwise flow development

In the following, we investigate the decay of the turbulence away from the array for the protocol with a transparency of $\phi =0.3$ and protocol spatial feature size of $129\textrm{mm}$ at maximum pump frequency. Our goal here is also to establish at what distance from the array the turbulence can be considered near homogeneous and isotropic. To this end, we perform velocity measurements along the centerline ($y=0\,\textrm{m},\;z=0\,\textrm{m}$) of the tunnel, with increasing distance away from the array.

**Fig. 21:**
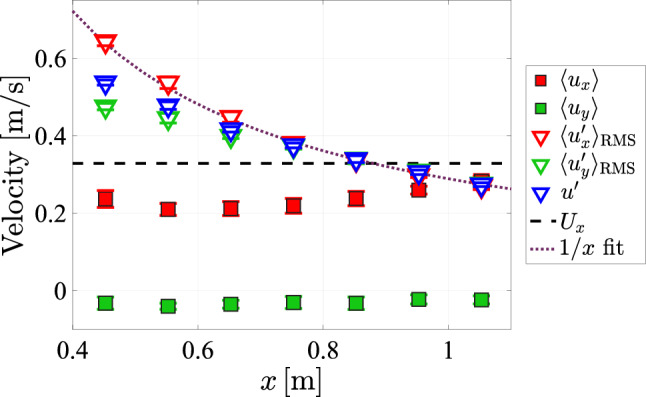
The mean and fluctuating velocity components plotted against *x* for an array transparency of $\phi =0.3$ and a pump frequency of $50\,\textrm{Hz}$, with the gray dashed line representing the estimated mean flow in the *x*-direction and the purple dotted Line representing a least squares fit of a 1/*x* velocity decay, fitted to the $\langle u'_x\rangle _{\textrm{RMS}}$ data

From Fig. 21 we can see that the mean streamwise velocity along the centerline slightly rises with increasing distance from the array. A possible explanation for this observation is that closer to the array ($x\sim 0.6\,\textrm{m}$) the velocity distribution is more likely to be spatially inhomogeneous in the mean since individual jets have not yet fully merged. Tan et al. (2023) state a critical distance of 5.5 times the array spacing (relating to a situation with all jets are firing, i.e., $\phi =1$) as the condition for homogeneous flow. At the present transparency setting of $\phi =0.3$, the effective array spacing, $M_{eff}=\sqrt{A_{T}/(\phi N_{\textrm{jets}})}\approx 7.8\,\textrm{cm}$ (the side length of a square that takes up $1/(\phi N_{\textrm{jets}})$ of the total cross-sectional area of the tunnel), is larger than the nominal value of 3.9cm, which makes it realistic that homogeneity is only attained at distances beyond $x\sim 0.6\,\textrm{m}$. The decay of the fluctuations of the vertical (*x*)-component conforms to the expected 1/*x* scaling of self-similar jets (Pope 2000; Tan et al. 2023) for the full range of *x* investigated.

Further, from our fit to the vertical fluctuating velocity component, shown in Fig. 21 by the purple dashed line, it is possible to obtain the product of the parameters $C_0$ and *B* from Eq. 25. We find that our fit corresponds to $C_0B=2.3$, which is higher than the value of $C_0B=1.5$ reported by Tan et al. (2023). It is likely that the discrepancy between our data and the data reported by Tan et al. (2023) originates from the difference in driving protocol. For the horizontal (*y*)-component, and thereby $u'$, deviations from this scaling occur, with lower values measured closer to the array ($x\le 0.7\,\textrm{m}$). As we observed in earlier mentioned measurements the velocity fluctuations are large in magnitude compared to the mean flow velocity, which means that the turbulence intensity is high.

**Fig. 22:**
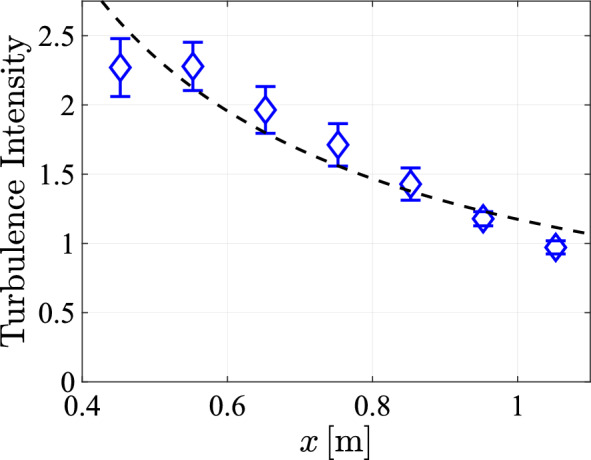
The turbulence intensity at $y=0$ with a transparency of $\phi =0.3$ and a pump frequency of $50\,\textrm{Hz}$, and a spatial feature size of $SFS=129\,\textrm{mm}$, plotted against *x*, and with the black dotted Line indicating a 1/*x* power law decay least squares fit

We have plotted the turbulence intensity against *x* in Figure 22. Near the array, turbulence intensities of more than 2 can be achieved. Following the 1/*x* decay of the fluctuations, also the turbulence intensity approximately decays with 1/*x* away from the array.

**Fig. 23:**
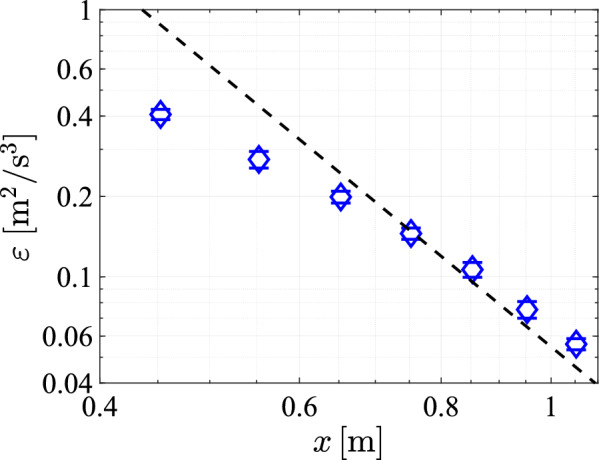
The dissipation rate, plotted against *x* at ${\text{y = 0}}\;{\text{cm}}$ for measurements with a transparency of $\phi =0.3$, a protocol spatial feature size of $129\;\textrm{mm}$ and a pump frequency of $50\,\textrm{Hz}$, with the black dashed line representing the fitted power law $\varepsilon \approx 0.055x^{-7/2}$

To further quantify the turbulence characteristics, we calculate the energy dissipation rate, as shown in Fig 23. Comparing these results to the energy dissipation rates achieved in the Twente Water Tunnel (Poorte and Biesheuvel 2002) ($0.007\,\mathrm {m^2/s^3}$) prior to its modification, as well as to rates reported in other setups, such as Variano and Cowen (2008) ($0.411\cdot 10^{-3}\,\mathrm {m^2/s^3}$), Masuk et al. (2019) ($0.16\,\mathrm {m^2/s^3}$), McCutchan and Johnson (2023) ($5.65\cdot 10^{-3}\,\mathrm {m^2/s^3}$), it is evident that ,following the modifications, our system achieves significantly higher energy dissipation rates in the Twente Water Tunnel. Moreover, these values are of the same order of magnitude as those observed in other jet systems.

Since the decay of the streamwise velocity fluctuations largely aligns with expectations and previous research, the stream wise development of the dissipation rate is also expected to align with previous results. As outlined in Tan et al. (2023), the dissipation rate is expected to scale as $\varepsilon \sim x^{-7/2}$ for turbulence decaying from a jet. However we observe in Figure 23 that the range in which the dissipation rate adheres to this power law is Limited. The deviation mainly occurs at distances smaller than 60 cm, which likely is still within the mixing region, where the power law is not expected to hold. Using the calculated dissipation rates and the mean fluctuation velocity, $u'$, we can calculate the Reynolds number for the Taylor microscale, as

26 $$\begin{aligned} \textrm{Re}_{\lambda }=\sqrt{\dfrac{15}{\nu }}\dfrac{u'^2}{\sqrt{\varepsilon }} \end{aligned}$$

**Fig. 24:**
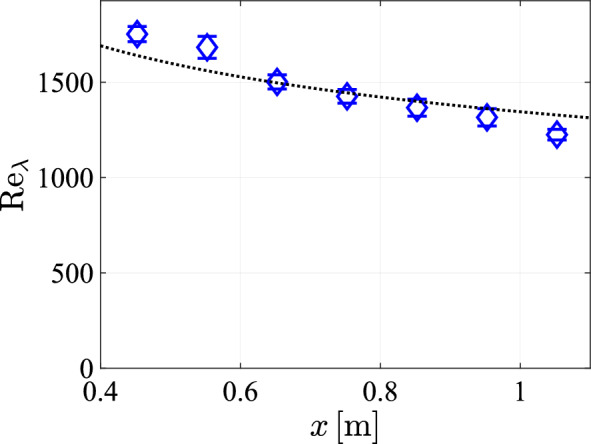
The Reynolds number of the Taylor microscale, plotted against *x* at $y=0\;cm$ with a transparency of $\phi =0.3$, a protocol spatial feature size of $129\textrm{mm}$ and a pump frequency of $50\,\textrm{Hz}$, and with the black dashed line indicating a least square fit of a $x^{-1/4}$ power law, such that $\textrm{Re}_{\lambda }\approx 1345x^{-1/4}$

We show the calculated values of $\textrm{Re}_{\lambda }$ in Fig. 24. The values exceed $Re_\lambda =1200$ at all distances from the array. Given the scaling relation $\varepsilon \sim x^{-7/2}$ for the energy dissipation rate and the scaling of the velocity fluctuations $u'\sim x^{-1}$, the Taylor Reynolds number is expected to scale as $\textrm{Re}_{\lambda }\sim x^{-1/4}$. A least squares fit corresponding to this scaling in Fig. 24, which yields $\textrm{Re}_{\lambda }\approx 1345x^{-1/4}$, is seen to approximate the data well.

#### Local homogeneity and isotropy

Due to the streamwise decay discussed in Sect. 4.2.4, homogeneity in this direction can only be approximate over shorter distances.

**Fig. 25:**
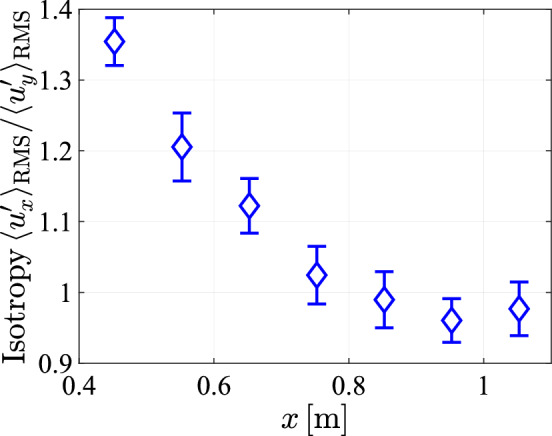
The isotropy as characterized by the ratio of the lateral and longitudinal velocity fluctuations, plotted against *x* at $y=0cm$

Here we focus on a region of about 5–10 cm around $x=0.95\textrm{m}$ in the streamwise direction on the centerline where the turbulence is still strong but decaying less rapidly. To investigate the in-plane flow statistics, we measured the flow velocity at 5 positions at equal height, laterally spaced with gaps of $5\;{\text{cm}}$.

**Fig. 26:**
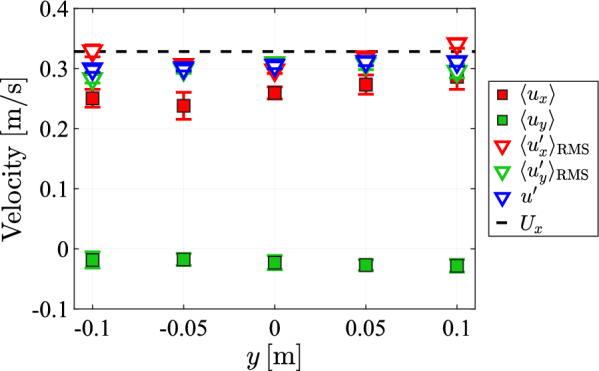
The mean and fluctuating velocity components at 5 lateral positions for a array transparency of $\phi =0.3$ at $x=0.95\textrm{m}$

The measurements presented in Fig. 26 indicate that the velocity statistics in the measured plane remain largely constant. In particular, both the mean and the variance of the streamwise velocity component are nearly unchanged as a function of *y*. The velocity fluctuations in the lateral direction vary slightly, with higher values further from the centerline of the tunnel.

**Fig. 27:**
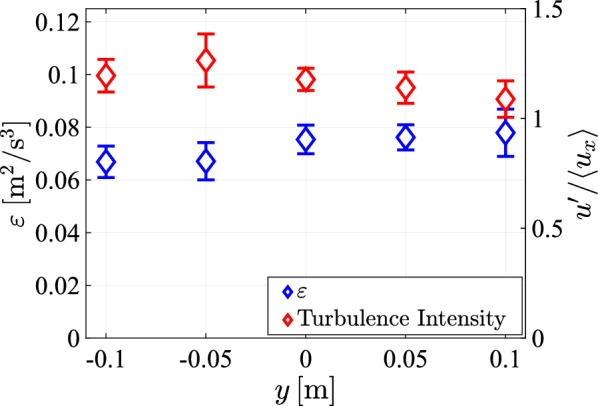
The energy dissipation rate and the turbulence intensity, plotted against *y* at $x = 95\;{\text{cm}}$, at maximum pump frequency and with a transparency of $\phi =0.3$

Also the turbulence intensity and energy dissipation rate in Fig. 27 remain approximately constant in the *y*-direction within the error margins of the measurements.

We conclude from these measurements that the flow conditions in the central $10\,\textrm{cm}$ in the horizontal direction of the tunnel can be considered homogeneous. As an indicator for the flow isotropy, the ratio between the velocity fluctuations in the streamwise and lateral directions is plotted in Fig. 25 as a function of the distance from the array. As discussed already in the context of Fig. 21, there is a high degree of anisotropy close to the array. This decays for larger *x*, with the velocity ratio decreasing to values around one, indicating isotropy, at $x \approx 70 \textrm{cm}$ and beyond. Deviations from one for $x \ge 70\textrm{cm}$ lie within the range reported in related studies (Hideharu 1991; Masuk et al. 2019; Ghazi Nezami and Johnson 2024; Poorte and Biesheuvel 2002) and the flow can be considered approximately isotropic in this region.

**Fig. 28:**
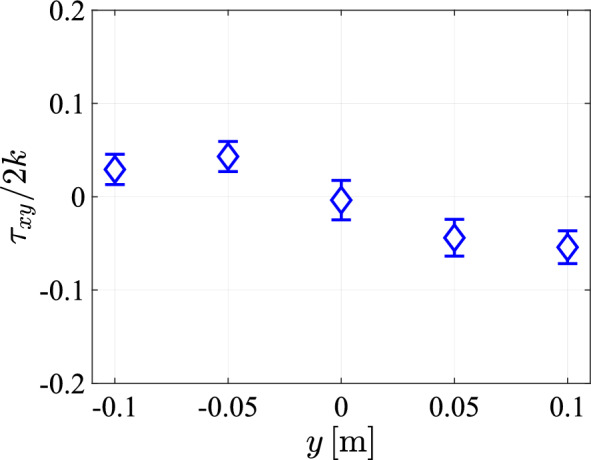
The *xy*-component of the Reynolds stress tensor normalized by twice the turbulence kinetic energy ($k=\frac{1}{2}(u')^2$), plotted at 5 lateral positions for a array transparency of $\phi =0.3$ and a pump frequency of $50\,\textrm{Hz}$ at $x = 0.95\;{\text{m}}$

**Fig. 29:**
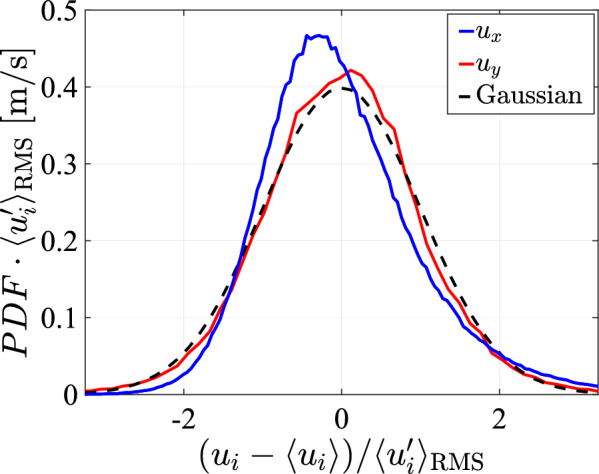
The probability density functions for the longitudinal and lateral velocity components at $y=0$ and $x=95\,\textrm{cm}$ with a transparency of $\phi =0.3$, a pump frequency of $50\,\textrm{Hz}$ and a protocol spatial feature size of $128\,\textrm{mm}$, compared with a Gaussian

As an additional measure for the isotropy of the flow, we plot the *xy*-component of the Reynolds stress tensor, calculated from the measurements of Fig. 26 and normalized by twice the turbulence kinetic energy, in Fig. 28 as a function of the lateral coordinate *y*. In an ideal isotropic turbulent flow, this component would remain zero. Deviations from zero remain below $5.5\%$ for the present measurements and is even much lower than that on the center line ($y = 0$), confirming the approximate isotropy of the flow. Finally, the normalized probability density functions for the longitudinal and lateral velocity components are plotted in Fig. 29 for a measurement at $y=0$ and $x=95\,\textrm{cm}$ below the array, with a transparency of $\phi =0.3$, a pump frequency of $50\,\textrm{Hz}$ and a protocol spatial feature size of $129\,\textrm{mm}$. The velocity distribution in the lateral direction is approximately Gaussian and symmetric around 0, but the longitudinal component is skewed toward high velocity events in the streamwise direction.

**Fig. 30:**
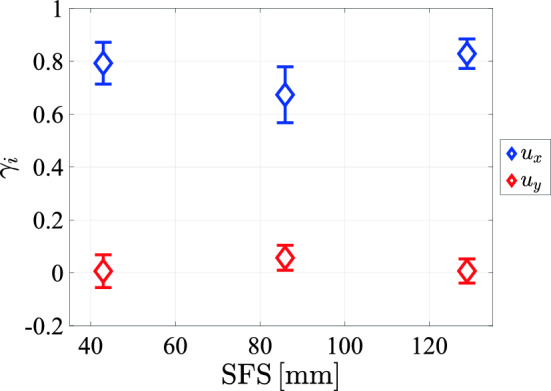
The skewness of the longitudinal and lateral velocity components at $y=0$ and $x=95\,\textrm{cm}$, plotted against the protocol spatial feature size with a transparency of $\phi =0.3$, and a pump frequency of $50\,\textrm{Hz}$

A similar degree of skewness is present in all measurements we performed, and it has also been observed in other setups using jet forcing (Tan et al. 2023; Pérez-Alvarado et al. 2016). A recent numerical study by Nguyen and Oberlack (2024) has shown that for a single jet, the longitudinal velocity distribution away from the jet centerline is skewed. While mixing of multiple jets is not present in that study, it indicates the possible inherency of skewness of the vertical velocity component in a jet system. To investigate if the skewness is protocol feature size dependent, we plot the skewness of the vertical and horizontal velocity components in Fig. 30 against the spatial feature size. These data show that the skewness is largely independent of the feature size and the data display no discernible trend. However, the magnitude of the skewness in the present setup falls within the range of reported values by Tan et al. (2023) and Pérez-Alvarado et al. (2016).

Due to the combined effect of the skewness in the vertical component, and the decaying turbulence, we cannot achieve ideal isotropy in our system, but we are able to create near homogeneous and isotropic turbulent conditions with powerful turbulence and horizontal in-plane homogeneity.

## Summary and outlook

We have constructed a jet array for the Twente Water Tunnel, which allows us to separate the method of turbulence creation from the background flow. To control the firing sequence of the jets, we developed a novel type of operating protocol based on 4D Open Simplex noise. We are able to tune the spatial and temporal coherence, and the percentage of active jets (the transparency) in these protocols. Our system is capable of producing high-intensity turbulence with turbulence intensities of Order 1, and dissipation rates of the order of $10^{-1}\mathrm {m^2/s^3}$ at $Re_\lambda \approx 1400$. It is possible to tune the characteristics of the turbulent flow by changing the power of the jet pump, the percentage of jets firing at any given moment, and the spatial distribution of the active jets in the tunnel. Our LDV measurements confirm local homogeneity and that our flow approaches isotropic conditions. As we indicated in Sect. 4, we had to limit the number of parameters we could investigate for this study. A further exploration of our system would include the varying of the remaining parameters that we have not adjusted, namely the temporal feature size and the applied background flow.The study of the effect of the background flow is mainly in the interest of further characterizing the flow conditions in our facility, as this topic has already been investigated previously, such as in Lee and Chu (2003) and Or et al. (2011). The temporal coherence of a protocol has been studied in previous work, notably in Variano and Cowen (2008) and in Pérez-Alvarado et al. (2016), where an increase in the "on-times" (the active periods) of the jets is seen to correspond to an increase in the magnitude of the velocity fluctuations. Part of these increases in the velocity fluctuations are hypothesized to be a direct result of the forcing scheme, rather than "true" turbulent fluctuations. We expect that changing the temporal coherence may have a similar effect in our jet array, especially for protocols with lower spatial feature sizes, since these are similar to previously investigated protocols. For larger spatial feature sizes we expect the effect to be similar, but due to the highly clustered nature of these protocols large differences may exist as in the currently presented experiments.

An important research topic to be investigated using our facility, is the dynamics of turbulence decay behind a jet array in a tunnel configuration. Recent research by Tan et al. (2023) has shown that turbulence decay behind a jet array is fundamentally different from decay from a passive or active array, and highlighted the importance of the jet nozzle diameter in this decay. The jet nozzles in our system can easily be exchanged to further test the role of the nozzle diameter. Installing nozzles with smaller diameters should facilitate faster homogeneity by allowing for higher transparencies without lowering the jet exit velocity, allowing for a further corroboration of the findings of Tan et al. (2023). Further, our research has shown that the scalings from the models developed by Tan et al. (2023) describe the streamwise decay of turbulence from a jet array well, but require further expansion to more accurately describe the flow from a jet array driven by a protocol with a non-uniform jet distribution. Experimental investigation into this subject in our system will help advance our understanding of this topic and provide additional data, which may reveal more about the universality and differences between jet array systems. Finally, with the now expanded parameter space, the jet array enables research into topics in multiphase turbulent flows, such as bubble-particle collisions and bubble deformation dynamics in strong turbulence.

## Figures and Tables

**Fig. 31:**
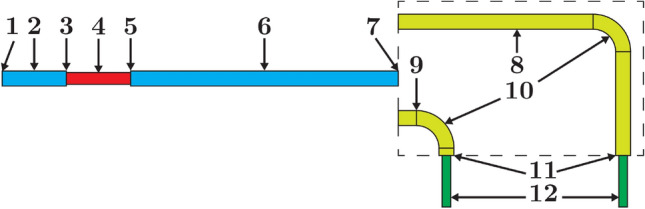
The schematic representation of the flow path between the manifold and the nozzle exit. Within the dashed rectangle, the shortest and longest grid tubes are shown

**Fig. 32:**
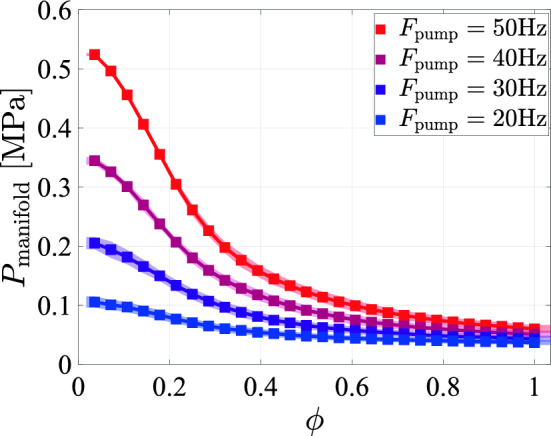
The recorded excess pressure in the grid manifolds for various transparencies and pump power settings, with the shaded areas indicating the standard deviations of the individual recordings

**Fig. 33:**
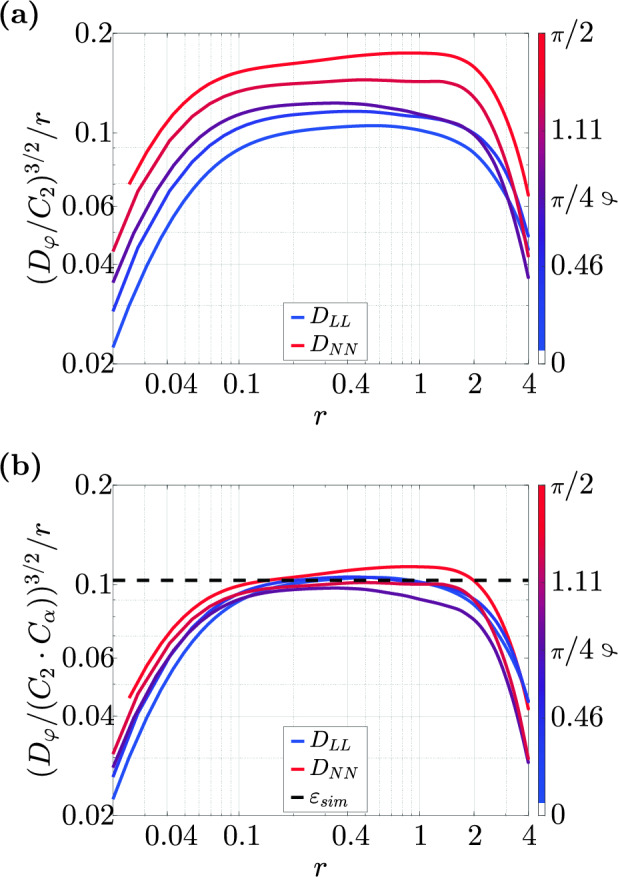
The calculated structure functions for displacements at 5 angles plotted against the displacement. **a** Shows the structure functions to the 3/2 power compensated only by $C_2^{3/2}$ and *r*, while **b** also includes the predicted scaling coefficient ($C_{\alpha }(\varphi )$

## Data Availability

The manifold pressure measurements (Figures 12 and 32) and all LDV measurement data (Figures 15, 16, 17, 18, 19, 20, 21, 22, 23, 24, 25, 26, 27, 28 and 29) are openly available from the 4TU.ResearchData repository at: 10.4121/0660fed3-f8ca-406d-872b-81aa819c1ba0. The data from Figures 5, 9, and 10 were generated from the protocol generation code, which is available at https://github.com/Dennis-van-Gils/project-TWT-jetting-grid

## Appendix A: Physical units of the feature sizes

The feature sizes *SFS* and *TFS* introduced in Sect. 3.2 are originally expressed as a multiple of the unit vector length of the OpenSimplex noise-space. Because the OpenSimplex implementation used here has a simplex grid spacing of 1 unit, its coherent features will also typically span a distance of around 1 unit. Hence, we can approximate their physical sizes as follows:

A1 $$\begin{aligned} SFS^{*} =\;&SFS \cdot \frac{\mathrm {PCS\_UNIT\_SI}}{\mathrm {PCS\_UNIT\_PX}},\end{aligned}$$

A2 $$\begin{aligned} TFS^{*} =\;&TFS \cdot \Delta t_{frame}, \end{aligned}$$

where $^{*}$ indicates that the variable is now bearing SI units. $\mathrm {PCS\_UNIT\_SI}$ is the physical distance in SI units corresponding to a unit increment of the protocol coordinate system (see Fig. 3(c)), for our facility equal to 27.5 mm. It is the half-distance between two valves on the *x* or *y*-axis. ${\mathrm {PCS\_UNIT\_PX}}$ is the number of pixels out of the noise frame that will be mapped to a unit increment of the protocol coordinate system (see Fig. 10a), for our facility set to 32 pixels. $\Delta t_{frame}$ is the time between consecutive frames, for our facility set to 0.05 s.

## Appendix B: Pressure loss model

For the estimation of the jet exit velocity, the pressure loss from the manifold to the nozzle exit needs to be estimated. This is done by calculating the pressure loss for individual segments of the plumbing between the nozzle exit and the manifold, and summing these to obtain the total estimated pressure loss. For the various sections of straight pipe, we use the Weisbach equation

B3 $$\begin{aligned} \Delta P = \frac{1}{2D}fL\rho U^2 \end{aligned}$$

where *D* is the hydraulic diameter, *f* is the Darcy-Weisbach friction factor, *L* is the length of the pipe section, $\rho$ is the fluid density, in this case water, and *U* is the bulk velocity of the fluid. We determine the bulk velocity of the fluid for a certain section *n* using

B4 $$\begin{aligned} U_n = U_{J}\dfrac{D_{J}^2}{D_n^2} \end{aligned}$$

where $U_{J}$ is the jet exit velocity, $D_{J}$ is the jet nozzle diameter, and $D_n$ is the diameter of the nth segment. We determine the friction factor by solving the Colebrook-White equation

B5 $$\begin{aligned} \dfrac{1}{\sqrt{f}}=-2\ln {\left( \dfrac{\varepsilon }{3.7D}+\dfrac{2.51}{\textrm{Re}\sqrt{f}}\right) } \end{aligned}$$

using a Newton solver. We list the relevant dimensions of the straight pipe sections in Table 1

**Table 1:** The dimensions and roughness for the various pipe segments used in the model

#	Segment	*L* [mm]	*D* [mm]	$\varepsilon \,\mathrm {[\mu m]}$
2	Manifold to Solenoid	60	14	0
4	Solenoid	60	11	0
6	Solenoid to tunnel wall	250	14	0
8	Tunnel wall to nozzle (long)	338	12	0
9	Tunnel wall to nozzle (short)	68	12	0
12	Nozzle	48	8	3

There are a variety of other types of segments in our system, shown schematically in Fig. 31, for which we calculate the pressure loss using

B6 $$\begin{aligned} \Delta P = K\cdot \frac{1}{2}\rho U^2 \end{aligned}$$

where *K* is the loss coefficient. We show the values for the loss coefficients of the various pipe segments in Table 2.

**Table 2:** The values of the loss coefficient for the various non-straight pipe flow obstructions

#	Segment	*K*	*D* [mm]	Reference
1	Entrance	0.5	14	Figure 8.24 Munson et al. (2012)
3	Solenoid contraction	0.15	11	Figure 8.26 Munson et al. (2012)
5	Solenoid expansion	0.18	14	Figure 8.27 Munson et al. (2012)
7	Tunnel wall contraction	0.45	12	Figure 8.26 Munson et al. (2012)
10	Grid tube elbow (*R* = 28 mm)	0.15	12	Figure 8.30 Munson et al. (2012)
11	Nozzle contraction	0.3	8	Figure 8.26 Munson et al. (2012)

We assume that the flow velocity inside the manifold is zero. Then the excess pressure in the manifold is

B7 $$\begin{aligned} P_{manifold}(U_{J}) = \sum _{n=1}^N\Delta P_n(U_{J}) + \frac{1}{2}\rho U_{J}^2 \end{aligned}$$

where $\Delta P_n$ is the pressure loss of the nth segment out of *N* ($N=11$ in our case).

Using Eq. B7 and the expressions for the pressure loss of each segment, the pressure loss between the manifold and the nozzle exit for a given jet velocity can be estimated.

To calculate the jet exit velocity for a given pressure, the expression needs to be inverted, which we do numerically. First we establish a range of jet velocities which that cover the actual value. Then we calculate the estimated pressure drop for these velocities. From the calculated set of jet velocities, we then select the velocity from the pressure–velocity pair that corresponds to the given pressure.

As indicated in Sect. 4 we measured the manifold pressures for various power and transparency settings, which we show in Fig. 32. The estimated jet exit velocities corresponding to those measurements are shown in Fig. 13.

To support the assumption that the flow velocity in the manifold is zero we perform a rough estimation of the flow velocity in the manifold, based on the flow rate out of the jet nozzles at $\phi =0.3$ and $F_{\textrm{pump}}=50\,\textrm{Hz}$, where we approximate the manifold as a pipe. Using the jet exit velocity estimate we obtain a flow velocity of $U_{\textrm{man}}\approx 0.8\,\mathrm {m/s}$ inside the manifold. The dynamic pressure resulting from this flow velocity is $P_{\textrm{dyn}}\approx 3\cdot 10^{-3}\,\textrm{MPa}$, which is much smaller than the measured manifold pressures. We therefore conclude that it is valid to neglect the dynamic pressure inside the manifold in the calculation of the jet exit velocity.

## Appendix C: Structure function coefficient

As mentioned in Sect. 4.2.1, the convection record method allows for the displacement *r* to be in any direction. Therefore the classical relations (Pope 2000)

C8 $$\begin{aligned} D_{LL} =\;&C_2\left( \varepsilon r\right) ^{2/3}\end{aligned}$$

C9 $$\begin{aligned} D_{NN}=\;&\frac{4}{3}C_2\left( \varepsilon r\right) ^{2/3} \end{aligned}$$

cannot be used directly, since the measured structure functions do not generally correspond to either the longitudinal or transverse structure functions. In this appendix we will derive the scaling coefficient for an arbitrary structure function, and provide evidence for its validity.

### C.1 Derivation

We start from the relation between this arbitrary structure function and the transverse and longitudinal structure functions, given by (see equation 6.25 in Pope (2000))

C10 $$\begin{aligned} \begin{aligned} D_{ij}(\vec {r},t)=\;&D_{NN}(r,t)\delta _{ij}+\left( D_{LL}(r,t)-D_{NN}(r,t)\right) \frac{r_ir_j}{r^2} \end{aligned} \end{aligned}$$

We are mainly interested in the single component structure functions for analysis, so Eq. C10 can be reduced to

C11 $$\begin{aligned} \begin{aligned} D_{ii}(r,t)=\;&D_{NN}(r,t)+\left( D_{LL}(r,t)-D_{NN}(r,t)\right) \frac{r_i^2}{r^2} \end{aligned} \end{aligned}$$

Taking the temporal average over a sufficiently long measurement time *T* eliminates the temporal dependence in Eq. C11 such that

C12 $$\begin{aligned} \begin{aligned} D_{ii}(r) =\;&\frac{1}{T}\int _0^TD_{NN}(r,t)dt +\frac{1}{T}\int _0^T\left( D_{LL}(r,t)-D_{NN}(r,t)\right) \cdot\frac{r_i^2}{r^2}dt. =\;\left\langle D_{NN}(r,t)\right\rangle _T +\Bigg \langle \left( D_{LL}(r,t)-D_{NN}(r,t)\right) \cdot&\frac{r_i^2}{r^2}\Bigg \rangle _T. \end{aligned} \end{aligned}$$

Given a sufficiently large domain over which the spatial averaging in the calculation of the structure functions occurs, the longitudinal and transverse structure functions should not depend on time. That makes Eq. C12

C13 $$\begin{aligned} \begin{aligned} D_{ii}(r) =\;&D_{NN}(r) +\Bigg \langle \left( D_{LL}(r,t)-D_{NN}(r,t)\right) \cdot\frac{r_i^2}{r^2}\Bigg \rangle _T \end{aligned} \end{aligned}$$

The second term in Eq. C13 can be written as

C14 $$\begin{aligned} \begin{aligned}&\left\langle \left( D_{LL}(r,t)-D_{NN}(r,t)\right) \frac{r_i^2}{r^2}\right\rangle _T=\left\langle \left( D_{LL}(r,t)-D_{NN}(r,t)\right) \right\rangle _T\cdot \left\langle \frac{r_i^2}{r^2}\right\rangle _T \end{aligned} \end{aligned}$$

assuming that the difference of the structure functions is independent of the direction of displacement, which is true under isotropic conditions. Due to the afformentioned time indepence of the longitudinal and transverse structure functions, the first term on the right hand side of Eq. C14 is equal to

C15 $$\begin{aligned} \begin{aligned}&\left\langle D_{LL}(r,t)-D_{NN}(r,t)\right\rangle _T=D_{LL}(r)-D_{NN}(r) \end{aligned} \end{aligned}$$

The second term of Eq. C14 can be calculated if the probability density function of the direction of displacement, $f_r(\Omega )$ which is a function of the solid angle, is known. Given a sufficiently long recording time, this function may be calculated from the recorded directions of displacement. Using this probability density function, the temporal average of equation C14 may be converted into an average over the solid angle, such that

C16 $$\begin{aligned} \left\langle \frac{r_i^2}{r^2}\right\rangle _T=\int _{\Omega }\frac{r_i^2}{r^2}f_{r}(\Omega )d\Omega . \end{aligned}$$

Introducing the results of Eqs. C15 and C16 into Eq. C13

C17 $$\begin{aligned} \begin{aligned} D_{ii}(r) =\;&D_{NN}(r) +\left( D_{LL}(r)-D_{NN}(r)\right) \cdot\int _{\Omega }\frac{r_i^2}{r^2}f_{r}(\Omega )d\Omega \end{aligned} \end{aligned}$$

We now define

C18 $$\begin{aligned} \alpha _i\equiv \int _{\Omega }\frac{r_i^2}{r^2}f_{r}(\Omega )d\Omega \end{aligned}$$

such that we can write Eq. C17 as

C19 $$\begin{aligned} D_{ii}(r) = (1-\alpha _i)D_{NN}(r)+\alpha _iD_{LL}(r) \end{aligned}$$

If we introduce the classical relations for the inertial sub range (Eqs. C8 and C9) into Eq. C19 it can also be written for the inertial sub range as

C20 $$\begin{aligned} D_{ii}(r) = (1-\alpha _i)\frac{4}{3}C_2(\varepsilon r)^{2/3}+\alpha _iC_2(\varepsilon r)^{2/3} \end{aligned}$$

or

C21 $$\begin{aligned} D_{ii}(r) = C_{\alpha i}C_2(\varepsilon r)^{2/3} \end{aligned}$$

with

C22 $$\begin{aligned} C_{\alpha i}\equiv \frac{4}{3}-\frac{1}{3}\alpha _i. \end{aligned}$$

Suppose that the displacement is exclusively in the *x* direction, which means that $D_{xx}=D_{LL}$ and $D_{yy}=D_{zz}=D_{NN}$. That would make $f_r(\Omega )=\delta _{\theta }(\theta -\pi /2)\delta _{\varphi }(\varphi )/\sin {(\theta )}$. The $\alpha _i$ values then become

C23 $$\begin{aligned} \begin{aligned} \alpha _x\!=\;&\!\int _0^{2\pi }\!\int _0^\pi \!\cos {(\varphi )^2}\sin {(\theta )^2}\\&\delta _{\theta }\left( \theta -\frac{\pi }{2}\right) \delta _{\varphi }(\varphi )d\theta d\varphi \!=\!1 \end{aligned} \end{aligned}$$

C24 $$\begin{aligned} \begin{aligned} \alpha _y\!=\;&\!\int _0^{2\pi }\!\int _0^\pi \!\sin {(\varphi )^2}\sin {(\theta )^2}\\&\delta _{\theta }(\theta -\pi /2)\delta _{\varphi }(\varphi )d\theta d\varphi \!=\!0 \end{aligned} \end{aligned}$$

C25 $$\begin{aligned} \begin{aligned} \alpha _z\!=\;&\!\int _0^{2\pi }\!\int _0^\pi \!\cos {(\theta )^2}\\&\delta _{\theta }(\theta -\pi /2)\delta _{\varphi }(\varphi )d\theta d\varphi \!=\!0 \end{aligned} \end{aligned}$$

which in turn give

C26 $$\begin{aligned} C_{\alpha x}=\;&1 \end{aligned}$$

C27 $$\begin{aligned} C_{\alpha y}=\;&C_{\alpha z}=4/3 \end{aligned}$$

and these are the exact values expected such that $D_{xx}=D_{LL}$ and $D_{yy}=D_{zz}=D_{NN}$, which means that in the limiting case of singular direction displacement, the coefficients revert to the values for the longitudinal and transverse structure functions.

### C.2 Coefficient validation

To test the validity of the derived scaling coefficient, we use a reference data set. We have sampled data from the JHU turbulence database, from the "Isotropic Turbulence on a $1024^3$ mesh" dataset (10.7281/T1KK98XB, Li et al. 2008). We use 3 centered planes (*XY*, *XZ* and *YZ*) of a full cross section at 41 time steps spaced at $\Delta t=0.25$s. From these cross sections, we calculate the structure functions from all 3 velocity vector components at 5 in-plane displacement angles $\varphi _{s}=[0,\arctan {(1/2)},\pi /4,\arctan {(2)},\pi /2]$. If we consider the structure functions of the *x*-component in the *XY* plane, the equation for $\alpha _{x}$ becomes

C28 $$\begin{aligned} \begin{aligned} \alpha _x=\;&\int _0^{\pi }\int _0^{2\pi }\cos {(\varphi )^2}\sin {(\theta )^3}\\ \cdot&\frac{1}{\sin {(\theta )}}\delta \left( \theta -\frac{\pi }{2}\right) \delta \left( \varphi -\varphi _s\right) d\theta d\varphi \end{aligned} \end{aligned}$$

where $f_{r}(\Omega )=\frac{1}{\sin {(\theta )}}\delta \left( \theta -\frac{\pi }{2}\right) \delta \left( \varphi -\varphi _s\right)$, which can be reduced to

C29 $$\begin{aligned} \alpha _x=\cos {(\varphi _s)^2} \end{aligned}$$

Due to symmetry, this expression for $\alpha _i$ is valid for all investigated planes, so $\alpha (\varphi )=\cos {(\varphi )^2}$ and therefore $C_{\alpha }(\varphi )=\frac{4}{3}-\frac{1}{3}\alpha (\varphi )$, which for the selected angles is equal to $C_{\alpha }=\left[ 1,\frac{16}{15},\frac{7}{6},\frac{19}{15},\frac{4}{3}\right]$. This allows us to combine the results for an angle from all planes. The results of these calculations are shown in Fig. 33a and b. It is clear from Fig. 33a that the structure functions are angle dependent, since there is a clear progression of the values of the structure functions from the low angles to the larger angles. Figure 33b further shows that when the predicted scaling coefficients are applied, the values converge on the expected value of the dissipation rate of the simulation. This shows that the structure functions are angle dependent in the way that we predict, thus serving as evidence to support the usage of the derived scaling coefficient.
